# An Asymmetrically Substituted Aliphatic Bis-Dithiolene Mono-Oxido Molybdenum(IV) Complex With Ester and Alcohol Functions as Structural and Functional Active Site Model of Molybdoenzymes

**DOI:** 10.3389/fchem.2019.00486

**Published:** 2019-07-11

**Authors:** Mohsen Ahmadi, Christian Fischer, Ashta C. Ghosh, Carola Schulzke

**Affiliations:** ^1^Institut für Biochemie, Universität Greifswald, Greifswald, Germany; ^2^Departement de Chimie Moléculaire, Université Grenoble Alpes, UMR CNRS 5250, Grenoble, France

**Keywords:** artificial molybdenum active site, aliphatic dithiolene, oxygen atom transfer, Moco model, Mo^IV^ oxo complex

## Abstract

A Mo^IV^ mono-oxido bis-dithiolene complex, [MoO(mohdt)_2_]^2−^ (mohdt = 1-methoxy-1-oxo-4-hydroxy-but-2-ene-2,3-bis-thiolate) was synthesized as a structural and functional model for molybdenum oxidoreductase enzymes of the DMSO reductase family. It was comprehensively characterized by *inter alia* various spectroscopic methods and employed as an oxygen atom transfer (OAT) catalyst. The ligand precursor of mohdt was readily prepared by a three-step synthesis starting from dimethyl-but-2-ynedioate. Crystallographic and ^13^C-NMR data support the rationale that by asymmetric substitution the electronic structure of the ene-dithio moiety can be fine-tuned. The Mo^IV^O bis-dithiolene complex was obtained by *in situ* reaction of the de-protected ligand with the metal precursor complex *trans*-[MoO_2_(CN)_4_]^4−^. The catalytic oxygen atom transfer mediated by the complex was investigated by the model OAT reaction from DMSO to triphenylphosphine with the substrate transformation being monitored by ^31^P NMR spectroscopy. [MoO(mohdt)_2_]^2−^ was found to be catalytically active reaching 93% conversion, albeit with a rather low reaction rate (reaction time 56 h). The observed overall catalytic activity is comparable to those of related complexes with aromatic dithiolene ligands despite the novel ligand being aliphatic in nature and originally perceived to perform more swiftly. The respective results are rationalized with respect to a potential intermolecular interaction between the hydroxyl and ester functions together with the electron-withdrawing functional groups of the dithiolene ligands of the molybdenum mono-oxido complex and equilibrium between the active monomeric Mo^IV^O and Mo^VI^O_2_ and the unreactive dimeric M${\text{o}}_{2}^{\text{V}}$O_3_ species.

## Introduction

**Figure 1:**
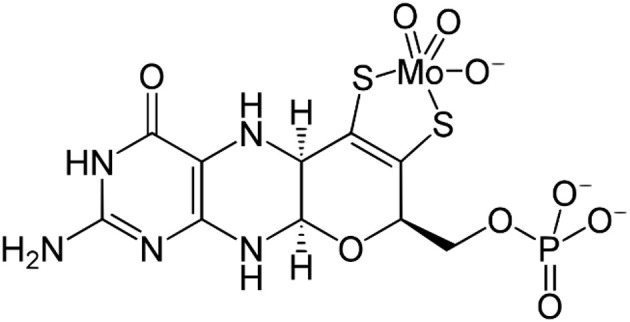
The chemical structure of the “free” biosynthesized molybdenum cofactor (Moco) prior to insertion into the apoproteins including the tricyclic molybdopterin ligand (MPT). Note: for XO and AO one oxido ligand will be replaced by a sulfido ligand before the active site is formed; in SO a cysteine binds molybdenum in the active site pocket.

Molybdenum dependent enzymes are essential contributors to the life of nearly every known organism on earth being it an ancient archaeon, a plant or a mammal which includes the modern human being (Mendel, 2007; Edwards et al., 2015). To date, four such molybdenum dependent enzymes have been discovered to be part of the human organism, which are sulfite oxidase (SO), xanthine dehydrogenase (XDH), aldehyde oxidase (AO), and mARC (Garner and Bristow, 1985; Hille, 2013; Hille et al., 2014; Schulzke and Ghosh, 2014). Defects in the maturation of the molybdenum cofactors (Figure 1), which can occur at different stages of the respective multistep biosynthesis, cause diseases (e.g., isolated sulfite oxidase deficiency: *i*SOD) due to the non-functioning of the molybdenum enzymes. This has consequences such as brain damage, motor retardation, convulsions etc. beginning right after birth and typically leading to infancy or early childhood death (Reiss, 2000). The extreme instability of the molybdenum cofactor prevents it from being biotechnologically produced and applied as treatment. Understanding exactly what makes Moco unstable and what makes it catalytically active is therefore of great interest for those aiming at developing an artificial cofactor which might be used as a respective drug in the future. This constitutes the motivation for our group and specifically for the study discussed in the following as one of many approaches. A moiety including molybdenum and one or two dithiolene ligands (representing molybdopterin—MPT; see Figure 1) is one of the most common motives in molybdenum cofactor bio-inorganic chemistry (Rajagopalan, 1997; Schulzke and Samuel, 2011).

**Scheme 1:**
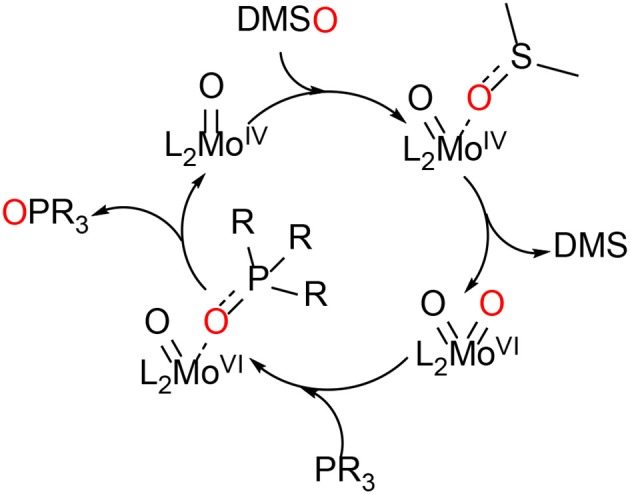
The model OAT reaction between DMSO and a phosphine catalyzed by a molybdenum bis-dithiolene complex (L = ene-dithiolate ligand; R = alkyl or aryl).

During the last 20 years, various bis-dithiolene mono-oxido molybdenum complexes have been developed and investigated (Donahue et al., 1998; Lim and Holm, 2001; Enemark et al., 2004; Döring et al., 2010; Schulzke, 2016; Ghosh et al., 2017). Such model compounds have helped understanding the roles of the dithiolene type ligands in the active sites of the DMSOR family enzymes, e.g., how they affect the electron and atom transfer reactivity during catalysis. Still, a comprehensive understanding of the roles of the different substituents is yet to be accomplished. During the catalytic reactions of these enzymes, molybdenum cycles between the oxidation states Mo^IV^ (d^2^) and Mo^VI^ (d^0^) constituting the fully reduced and fully oxidized active species. The oxidation state Mo^V^ (d^1^) is part of the regeneration of the active site by two proton coupled electron transfer steps (PCET). Dithiolenes are *non-innocent* ligands which can affect the electronic structure of their molybdenum (and tungsten) complexes by providing the central metal with electron density shifted from a sulfur p-orbital bearing a lone pair to an empty metal d-orbital by respective orbital overlap or even by full ligand to metal charge transfer (LMCT) (Kirk et al., 2004; Sugimoto et al., 2009). Although the role of molybdenum in the DMSOR enzymes for the catalysis of the oxygen atom transfer reactions (OAT) is quite well-understood, the role of the molybdopterin ligand (MPT) remains to be comprehensively deciphered. The synthesis of MPT or any artificial close relative of it represents a major chemical challenge and the respective attempts are still ongoing in a small number of research groups, although some significant advances have already been reported (Bradshaw et al., 1998, 2001a; Sugimoto et al., 2005; Williams et al., 2012, 2015; Basu and Burgmayer, 2015; Gisewhite et al., 2018). Holm and coworkers have not only developed OAT model reactions relevant for the molybdenum enzymes' interconversion but have also extensively reviewed them already in the 1980's (Berg and Holm, 1985; Holm, 1987). In many model reactivity studies dimethyl sulfoxide (DMSO) was employed for the oxidation of Mo^IV^O complexes, which is a natural substrate of DMSO reductase, and organic phosphines (PR_3_, as easy to handle *non*-natural co-substrates) were used for the reduction of Mo^VI^O_2_, Scheme 1.

Both, [Mo^IV^O(dt)_2_] as well as [Mo^VI^O_2_(dt)_2_] complexes (*dt* = dithiolene ligand) employing distinct dithiolenes were reported by us before and shown to be active catalysts for the OAT reaction with varied capabilities (Ghosh et al., 2017). What became apparent from many studies from others as well as our own, was the detrimental influence of aromatic dithiolenes on the catalytic performance, in particular of those in which the ene of the dithiolene is actually part of the aromatic moiety, e.g., in benzenedithiolate (Fischer and Fischer, 2017). Aliphatic dithiolene ligands, in contrast, have proven to be much more instable species and consequently also much better catalysts due to the higher activity. Introduced here are now a new aliphatic dithiolene ligand and its Mo^IV^O bis-dithiolene complex. Both were characterized comprehensively as were all ligand precursors. The IR and UV-vis spectroscopic data of the complex were compared to known data of related compounds and the complex' ability to catalyze OAT reactions was investigated. The observed surprisingly poor performance is discussed referring to (i) the presence of specific substituents (ester and alcohol groups), (ii) crystallographic and spectroscopic data revealing *inter alia* information about bond lengths and strengths, (iii) substrate formation monitoring, and (iv) probable intermolecular interactions.

## Experimental

### Synthetic Procedures

All reactions and manipulations were carried out using standard Schlenk and glove box techniques under an atmosphere of high purity nitrogen (Schlenk) or argon (glove box). All solvents were dried, distilled and either degassed or purged with dinitrogen or argon prior to use. Ethylene trithiocarbonate (Kim et al., 2008) and the molybdenum precursor K_3_Na[MoO_2_(CN)_4_]·6H_2_O (Smit et al., 1993) were synthesized according to previously reported procedures.

### Dimethyl 2-Thio-1,3-Dithiole-4,5-Dicarboxylate (1)

In a modification of a literature procedure (Easton and Leaver, 1965) dimethyl but-2-ynedioate (18.3 mmol, 2.25 mL) and ethylene trithiocarbonate (18.3 mmol, 2.52 g) were heated to reflux for 10 h under N_2_ in anhydrous toluene. The solution was left to cool to r.t. and filtered. The remaining solution was kept at −20°C and adding *n*-hexane to the solution led to precipitation of yellowish crystalline compound **1**. Yield: 3.8 g, 85%. ^1^H NMR (CDCl_3_, 300 MHz): δ (ppm): 3.90 (s, 6H, CH_3_). ^13^C NMR (CDCl_3_, 75 MHz): δ (ppm): 207.2 (C=S), 157.9 (C=O), 138.1 (C=C), 53.85 (CH_3_). FT–IR bands (KBr pallet, cm^−1^): 3446 (br), 2954 (s), 2918 (w), 1745 (s), 1720 (s), 1552 (s), 1257 (br), 1101 (s), 1087 (m), 1060 (s), 1008(s), 993(s), 921(s), 837 (w), 777(w), 761(w), 744(w), 698(w), 511 (m). APCI-MS (EI): m/z calculated for C_7_H_6_O_4_S_3_: 249.94; Found: 250.71 [M+H^+^]. Elemental analysis for C_7_H_6_O_4_S_3_: calc. (%): C, 33.59; H, 2.42; S, 38.43. Found: C, 34.65; H, 2.46; S, 37.38.

### Methyl 5-(Hydroxymethyl)-2-Thioxo-1,3-Dithio-4-Carboxylate (2)

**Scheme 2:**
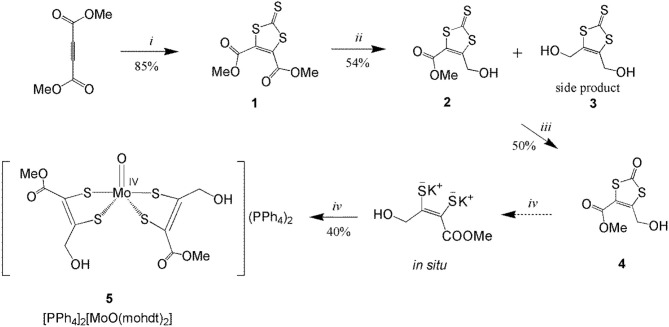
Synthesis of compounds **1**–**4** and molybdenum complex **5**. (*i*) ethylene trithiocarbonate, toluene, reflux, 10 h; (*ii*) 2 eq. NaBH_4_, dry LiCl, (anhydrous THF:EtOH), −15°C to 0°C, 1 h; (*iii*) Hg(OAc)_2_, AcOH/CHCl_3_, 6 h; (*iv*) KOH, degassed MeOH, [MoO_2_(CN)_4_]K_3_Na, degassed H_2_O, Ph_4_PCl salt, under N_2_, 50°C, 3 h.

To a well-stirred solution of **1** (3.62 g, 14.5 mmol) and dry LiCl (1.22 g, 29 mmol) in anhydrous THF (40 mL) and EtOH (15 mL) at −15 to −10°C powdered sodium borohydride (NaBH_4_, 1.15 g, 30.5 mmol) was slowly added in small portions over a duration of 20 min. An exothermic reaction took place and the temperature was kept under −10°C at all times and for further 30 min. Then H_2_O (150 mL, 0°C) was added followed by concentrated aqueous HCl (4 N, carefully and portion-wise) until the evolution of H_2_ gas ceased. The mixture was extracted with EtOAc (3 × 100 mL), and the extract was dried over Na_2_SO_4_. Evaporation of the solvent gave a yellow oily residue which was re-dissolved in CH_2_Cl_2_/EtOAc (2:1, 25 mL) and purified by column chromatography. The first yellow fraction contained trace amounts of the starting material and the second fraction contained the mono-alcohol. The second fraction was concentrated *in vacuo* to give brownish-yellow crystalline compound **2** (see Scheme 2). Yield: 1.6 g, 54%. ^1^H NMR (CDCl_3_, 300 MHz): δ (ppm): 4,94 (s, 2H, CH_2_), 3.88 (s, 3H, CH_3_). ^13^C NMR (CDCl_3_, 75 MHz): δ (ppm): 210.7 (C=S), 163.6 (C=O), 158.6 (CO–C=C), 124.82 (CH_2_–C=C), 60.5 (CH_2_), 52.9 (CH_3_). FT–IR bands (KBr pallet, cm^−1^): 3446 (br), 3012 (w), 2951 (s), 2924 (w), 2017 (br), 1994 (br), 1745 (s), 1718 (m), 1627 (m), 1618 (m), 1550 (m), 1435 (s), 1261 (br), 1070 (s), 758 (s), 599 (w), 514 (w), 460 (m). APCI-MS (EI): m/z calculated for C_6_H_6_O_3_S_3_: 221.95; Found: 222.8 [M+H^+^]. Elemental analysis for C_8_H_10_O_4_S_3_ (1/2 × EtOAc as co-crystallized lattice solvent) calc. (%): C, 36.07; H, 3.71; S, 36.11. Found: C, 36.31; H, 3.32; S, 36.16. The side product (4,5-bis(hydroxymethyl)-1,3-ene-dithio-2-thione (**3**, di-alcohol) was collected from the third fraction by column chromatography as yellow needle shaped microcrystalline solid (see Scheme 2). ^1^H NMR (CD_3_OD, 300 MHz): δ (ppm): 4.52 (s, 2H, CH_2_). ^13^C NMR (CD_3_OD, 75 MHz): δ (ppm): 214.4 (C=S), 143.5 (C=C), 57.8 (CH_2_). FT–IR bands (KBr pallet, cm^−1^): 3421 (br), 2953 (s), 1982 (br), 1718 (br), 1436 (m), 1361 (w), 1327 (w), 1247 (m), 1201 (m), 1180 (m), 1074 (m), 1053 (s), 1035 (m), 991 (s), 635 (m), 518 (m). APCI-MS (EI): m/z calculated for C_5_H_6_O_2_S_3_: 193.95; Found: 194.80 [M+H^+^].

### 4-Methyl-Carboxylate-5-Hydroxymethyl-1,3-ene-Dithio-2-One (4, mohdtC=O)

Four equivalents of mercury acetate, Hg(OAc)_2_ (7 g, 21.8 mmol) were added to a stirred solution of **2** (1 g, 5 mmol) in 100 mL AcOH/CHCl_3_ (2:1) for 6 h. The reaction was followed by TLC (silica, DCM). The resulting pale green mixture was filtered through a Celite pad to remove the mercury salts (mainly HgS). The resulting solution was washed first with water and then with aqueous NaHCO_3_ and dried over Na_2_SO_4_. The final light yellowish powder was collected after short silica column chromatography (DCM/EtOAc; 3:1). Yield: 0.5 g, 50%. ^1^H NMR (CDCl_3_, 300 MHz): δ (ppm): 4.93 (s, 2H, CH_2_), 3.86 (s, 3H, CH_3_). ^13^C NMR (CDCl_3_, 75 MHz): δ (ppm) = 188.7 (C=O_oxo_), 160.2 (C=O_COOMe_), 151.6 (CO–C=C), 117.6 (CH_2_–C=C), 60.1 (CH_2_), 53.1 (CH_3_). FT–IR bands (KBr pallet, cm^−1^): 3483 (br), 2956 (w), 1701(s), 1654 (s), 1618 (m), 1544 (m), 1435 (s), 1352 (m), 1286 (br), 1220 (m), 1068 (m), 1029 (w), 970 (w), 952 (w), 894 (w), 813 (w), 759 (w), 607 (w), 470 (w). APCI-MS (EI): m/z calculated for C_6_H_6_O_4_S_2_: 205.97; Found: 206.80 [M+H^+^]. Elemental analysis for C_6_H_6_O_4_S_2_: calc. (%): C, 34.94; H, 2.93; S, 31.09. Found: C, 35.01; H, 2.95; S, 30.52. Electronic absorption spectral data in CH_3_CN (λ_max_, nm (ε/M^−1^ cm^−1^)): 211 (2682), 285 (br, 2340).

### [Ph_4_P]_2_[MoO(mohdt)_2_] (5)

The ligand precursor **4** (0.12 g, 0.6 mmol) was added to a Schlenk flask containing 16 mL of 0.1 M KOH solution in anhydrous methanol under N_2_ atmosphere and stirred for 2 h. The solution turned light yellow and to this a blue solution of K_3_Na[MoO_2_(CN)_4_]·6H_2_O (0.15 g, 0.3 mmol) dissolved in 8 mL degassed water was added by cannula under N_2_. The reaction mixture was stirred at 50°C for 3 h. Then 0.21 g of tetraphenylphosphine chloride, Ph_4_PCl dissolved in 8 mL degassed water was added to the reaction mixture. The final red solution was concentrated in vacuum to dryness. It was then dissolved in 40 ml of CH_3_CN and the residue was filtered off. The organic solution was transferred to another Schlenk flask and anhydrous diethyl ether was added slowly. The brownish-red precipitate was collected and dried under reduced pressure. Yield: 0.3 g, 40%. ^1^H NMR (CD_3_CN, 300 MHz): δ (ppm): 7.80-8.93 (m, 4H, Ph_4_P^+^), 7.51-7.75 (m, 16H, Ph_4_P^+^), 4.57 (s, 4H, CH_2_), 3.66 (s, 3H, CH_3_). ^13^C NMR (CD_3_CN, 75 MHz): δ (ppm): 165.2 (CO), 152.8 (CO–C=C), 136.35, 135.7, 135.5, 131.3, 131.2, 119.4 (CH_2_–C=C), 63.7 (CH_2_), 54.7 (CH_3_). FT–IR bands (KBr pallet, cm^−1^): 3431 (br), 3055 (w), 3022 (w), 2924 (s), 1718 (br), 1585 (m), 1541(s), 1483 (s), 1436 (s), 1330 (br), 1228 (s), 1188 (w), 1165, 1109 (s), 1026 (w), 997 (s), 977 (s), 925 (s), 885 (s), 758 (s), 723 (s), 688 (s), 615 (w), 526 (s), 459 (w). MALDI-TOF-MS (Negative ion linear mode using 2,5-dihydroxybenzoic acid, DHB as matrix): m/z calculated for C_10_H_12_MoO_7_S_4_: 469.85, Found: 469.26. Elemental analysis for C_58_H_54_MoO_7_P_2_S_4_: calc. (%): C, 60.62; H, 4.74; S, 11.16. Found: C, 60.70; H, 4.37; S, 11.10. Electronic absorption data in CH_3_CN (λ_max_, nm (ε = M^−1^ cm^−1^)): 225 (10653), 256 (sh, 2563), 265 (2758), 277 (2340), 323 (1023).

### Physical Measurements

NMR measurements were recorded on a Bruker Avance II-300 MHz instrument. All samples were dissolved in deuterated solvents and chemical shifts (δ) are given in parts per million (ppm) using solvent signals as reference (CDCl_3_^1^H: δ = 7.24 ppm; ^13^C: δ = 77.0 ppm; CD_3_OD ^1^H: δ = 3.31, 4.87 ppm; ^13^C: δ = 49.15 ppm, CD_3_CN ^1^H: δ = 1.94 ppm; ^13^C: δ = 1.3 ppm) related to external tetramethylsilane (δ = 0 ppm). Spectra were obtained at 25°C unless otherwise noted. Coupling constants (*J*) are reported in Hertz (Hz) and splitting patterns are designated as s (singlet), d (doublet), t (triplet), q (quartet), quint (quintet), sext (sextet), m (multiplet), dd (doublet of doublet). Infrared spectra were recorded as KBr disks in the range 4000–400 cm^−1^ on a PerkinElmer Fourier-Transform Infrared (FT–IR) spectrophotometer. The assignment of the bands was done with subjective appreciation: w, weak; m, medium; s, strong; vs, very strong; br, broad. UV/Vis spectra were recorded on a Shimadzu UV-3600 spectrophotometer. Elemental analyses (C, H, N and S) were carried out with an Elementar Vario Micro Cube elemental analyzer. Mass spectra of organic molecules (APCI) were recorded with the high performance compact mass spectrometer Advion Expression CMS. Resolution: 0.5–0.7 m/z units (FWHM) at 1,000 m/z units sec^−1^ over the entire acquisition range. For compound **5** the mass spectra were measured on a Bruker microflex matrix assisted laser desorption/ionization (MALDI-TOF) spectrometer and the Advion Expression CMS spectrometer.

Electrochemical measurements were carried out with an AUTOLAB PGSTAT12 potentiostat/galvanostat using a glassy carbon working electrode with a reaction surface of 1 mm^2^ in acetonitrile solution with 0.1 M of [*n*Bu_4_N][PF_6_] as supporting electrolyte. A platinum knob electrode (together with internal referencing vs. ferrocene/ferrocenium; F_c_/${\text{F}}_{\text{c}}^{+}$) was used as reference electrode and a platinum rod electrode as auxiliary electrode. All measurements were controlled with the NOVA software and carried out inside a glove box under argon atmosphere.

### X-Ray Crystallography

Suitable single crystals of compounds **1**, **2**, **3**, and **4** were mounted on a thin glass fiber coated with paraffin oil. X-ray single-crystal structural data were collected at low temperature (170 K) using a STOE-IPDS II diffractometer equipped with a normal-focus, 2.4 kW, sealed-tube X-ray source with graphite-mono-chromated MoK_α_ radiation (λ = 0.71073 Å). The program XArea was used for integration of diffraction profiles; numerical absorption correction was made with the programs X-shape and X-red32; all from STOE. The structure was solved by SIR92 (A. Altomare et al., 1993) or SHELXL-2013 (Sheldrick, 2008) and refined by full-matrix least-squares methods using SHELXL-2013 or SHELXL-2016 (Sheldrick, 2008, 2015). The non-hydrogen atoms were refined anisotropically. The oxygen bound alcohol hydrogen atoms in **2**, **3** and **4** were refined freely. All other hydrogen atoms were refined isotropically on calculated positions using a riding model with their U_iso_ values constrained to 1.5U_eq_ of their pivot atoms for methyl and hydroxyl groups and to 1.2U_eq_ for all other C-H bonds. All calculations were carried out using SHELXL-2013/16 and the WinGX system, Ver2014.01 (Farrugia, 2012). Crystallographic data were deposited with the Cambridge Crystallographic Data Centre, CCDC, 12 Union Road, Cambridge CB21EZ, UK. These data can be obtained free of charge on quoting the depository numbers CCDC (**1**) 1858446, (**2**) 1858448, (**3**) 1858449, and (**4**) 1858447 by FAX (+44-1223-336-033), email (deposit@ccdc.cam.ac.uk) or their web interface (at http://www.ccdc.cam.ac.uk). Crystal and refinement data are summarized in Table 1. Details of crystal structural data are tabulated in the electronic supporting information (Tables S1–S18).

**Table 1:** Crystal and refinement data for **1**, **2**, **3**, and **4** at 170 K.

	**1**	**2**	**3**	**4**
Formula	C_7_H_6_O_4_S_3_	C_5_H_6_O_2_S_3_	C_6_H_6_O_3_S_3_	C_6_H_6_O_4_S_2_
Mw	250.30	194.28	222.29	206.23
Crystal system	Monoclinic	Monoclinic	Monoclinic	Monoclinic
Space group	*P*2_1_/*c*	*P*2_1_/*c*	*P*2_1_/*c*	*P*2_1_/*n*
a [Å]	13.209(3)	9.6501(19)	12.653(3)	3.8936(8)
b [Å]	9.4789(19)	11.081(2)	4.5334(9)	9.1518(18)
c [Å]	8.1022(16)	14.588(3)	15.650(3)	22.568(5)
β [°]	102.27(3)	97.81(3)	94.58(3)	93.07(3)
d_calc_ [mg/m^3^]	1.677	1.670	1.650	1.706
Z	4	8	4	4
V [Å^3^]	991.3(4)	1545.5(5)	894.8(3)	803.0(3)
μ [mm^−1^]	0.730	0.891	0.790	0.631
reflns collected/unique	10584/2666	9074/3238	9059/2421	7550/2142
reflns/R_int_	0.0882	0.0759	0.0674	0.0488
${\text{R}}_{1}^{\text{a}}$ (w${\text{R}}_{2}^{\text{b}}$) (I>2σ(I))	0.0405 (0.0771)	0.0357 (0.0589)	0.0635 (0.1628)	0.0336 (0.0730)
GOF [F^2^]	0.988	0.894	0.966	1.034
residual density [Å^−3^]	0.262/−0.372	0.294/−0.352	0.536/−0.475	0.320/−0.371

a *R*_1_ = ∑||*F_o_*|−|*F_c_*||/∑|*F_o_*|.

b *R_w_ = [∑{w(F${}_{\text{o}}^{\text{2}}$- F${}_{\text{c}}^{\text{2}}$)^2^}/∑{w(F${}_{\text{o}}^{\text{2}}$)^2^}]^1/2^*.

## Results and Discusion

### Syntheses

The synthetic route to dithiolene ligand precursors **1**–**4** along with the complexation reaction are displayed in Scheme 2. The ligand mohdt is readily obtained by a three-step procedure starting from dimethyl but-2-ynedioate. Trithiocarbonate **1** was synthetized by reaction of the symmetrical alkyne with ethylene trithiocarbonate in anhydrous toluene under reflux conditions in a modified literature procedure (Easton and Leaver, 1965). Compound **1** was then reduced by little more than two equivalents of sodium borohydride (NaBH_4_) in the presence of LiCl and dry THF/EtOH similar to procedures applied previously (Jeppesen et al., 1999; Bellanger et al., 2012) but in distinct stoichiometry as a different, asymmetric compound was targeted here. The reduced unsymmetrical trithiocarbonate **2** was collected as major compound after column chromatography. Also a small amount of symmetrically substituted compound **3** (which was the target in the two reports cited above) as side product could be isolated and identified suggesting that the stoichiometric amount of NaBH_4_ needs to be controlled very carefully in this reduction reaction.

An often applied procedure for the synthesis of oxo-dithiocarbonate compounds is oxidizing the thione moiety in trithiocarbonate backbones with mercury acetate in the presence of acetic acid utilizing mercury's thiophilicity (Nguyen et al., 2010). Compound **2** was oxidized accordingly and then purified by column chromatography yielding the final dithiolene precursor **4**, mohdtC=O (see Scheme 2).

A detailed comparison of infrared, ^1^H and ^13^C NMR spectroscopic data of the trithiocarbonates (**1**, **2**, and **3**) and the dithiocarbonate **4** reveals some interesting aspects of the C=S, C=O and C=C functional groups. The C=S signals in the ^13^C NMR spectra of **1**, **2** and **3** were observed at δ 207.2, 210.8, and 214.4 ppm, respectively, which differs as expected from the C=O signal of **4** at δ 188.7 ppm (see Supplementary Material). The differences in the ^13^C NMR spectra for the C=C bonds in **1** (138.1 ppm; ester only), **2** (158.6 and 124.8 ppm; mixed) and **3** (143.5 ppm, hydroxyl only) are predominantly due to the presence (or absence) of the electron-donating and -withdrawing ester and hydroxyl functions, respectively. Most notably, in the unsymmetrically substituted **2** the comparable downfield and upfield shifts are more pronounced than in the symmetric compounds indicating a considerable push-pull effect induced by the asymmetry (see also the discussion in the structural characterization part below). Changing the C=S (**2**) to a C=O (**4**) function results in an upfield shift by ca. 7 ppm for both carbon atoms of the C=C moiety (151.6 and 117.6 ppm, mixed). The ^1^H NMR spectrum of **4** in CDCl_3_ displays two singlets at δ = 3.86 and 4.93 ppm, assigned to the methyl and methylene protons, respectively, which is almost identical to the values obtained for **2**. This indicates that an exchange of C=O for C=S has no effect on these protons. Compared to the symmetric species the methylene protons are shifted downfield by ca. 0.4 ppm and the methyl protons are shifted upfield by an average of 0.03 ppm. The C=S stretching frequencies in the IR spectra for **1**, **2**, and **3** were observed at 1067, 1070, and 1053 cm^−1^ (Liu et al., 2010), respectively, while the C=O_oxo_ frequency in **4** is found at 1,654 cm^−1^ in accordance with the generally stronger bond between C and O (see Supplementary Material).

Complex **5** was synthesized according to a modified method already reported in the literature (Bradshaw et al., 2001b). The elemental analysis, the infrared, electronic absorption and NMR spectroscopic data, and the MALDI-TOF mass spectrometric data of **5** unambiguously support the formation of the mono-oxido Mo^IV^ center coordinated by two {S_2_C_2_(CO_2_Me)(CH_2_OH)}^2−^ ligands. A comparison with known data of a closely related compound from the literature (Coucouvanis et al., 1991) and the similarity of the respective analyses further validates the proposed chemical structure of the complex. The molecular ion peak of **5** was detected at m/z 469.3 by MALDI-TOF-MS in the negative ion linear mode using 2,5-dihydroxy benzoic acid (DHB, 10 mg/mL in acetonitrile/water mixture (1/1, v/v) containing 0.1% TFA) (see Supplementary Material). The molecular ion peak of complex **5** was also detected by ESI-MS (-) analysis with a fitting isotopic pattern at m/z 460.2 to 469.1. The tetraphenylphosphonium counter cations (PP${\text{h}}_{4}^{+}$) were observed at m/z 339.0 in the positive ESI-MS mode (see Supplementary Material).

**Figure 2:**
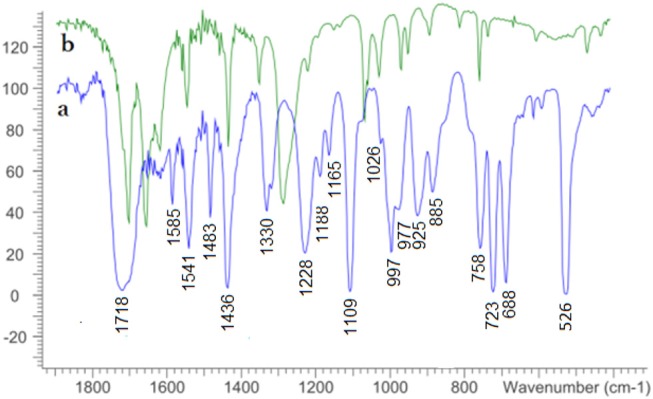
Solid state FT-IR spectra of complex **5** (a, bottom) and ligand precursor **4** (b, top) measured as KBr pellets.

The ^13^C-NMR signals for the C=C bond in **4** are slightly shifted to the downfield/deshielded/higher frequency region in complex **5**, which is a characteristic difference between a free dithiolene ligand precursor and the de-protected ene-dithiolate ligand coordinated to a Mo^IV^O center. The π-delocalization within the dithiolene and the charge donation to the metal can be assessed considering the frequency of the C=C stretching mode in the FT-IR spectrum typically found in a range of 1,400–1,600 cm^−1^ as the C=C bond weakens with increased donation to the metal (Garton et al., 1997). A tentative assignment of the band at 1,541 cm^−1^ to this vibration, which is only marginally shifted from 1,544 cm^−1^, supports the presence of the ene-dithiolate rather than reduction of the metal with concomitant oxidation of the ligand to a radical species (partial thione character). The M=O stretching frequency of **5** at 925 cm^−1^ exhibits a substantial shift from the Mo precursor 728 cm^−1^ (Figure 2) (Ghosh et al., 2017). This is comparable to reported related Mo^IV^O complexes such as [MoO{S_2_C_2_(COOMe)_2_}_2_]^2−^ (Coucouvanis et al., 1991), [MoO{S_2_C_2_(CN)_2_}_2_]^2−^ (Donahue et al., 1998) and [MoO{S_2_C_2_(CONH_2_)_2_}_2_]^2−^ (Oku et al., 1997). The IR spectrum of mohdtC=O (**4**) further shows two sharp bands at 1,710 cm^−1^, at 1,654 cm^−1^ and one medium signal at 1,618 cm^−1^ belonging to (C=O)_ester_ and (C=O)_oxo_ dithiolene stretching frequencies, respectively. The (C=O)_ester_ vibration is shifted to higher frequency in complex **5** (ν(C=O)_ester_: 1,718 cm^−1^) and (C=O)_oxo_ has disappeared after complexation as expected (see Figure 2). Further C–O and C–S frequencies are difficult to identify/assign as they are masked by the dominating C–H stretching bands of the Ph_4_P^+^ counter-cation in the region 688–758 cm^−1^ (Tchouka et al., 2011).

**Figure 3:**
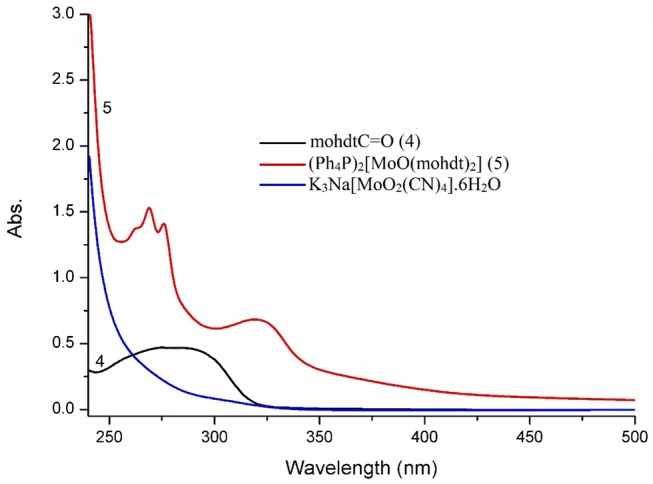
Comparison of UV-vis spectra of mohdtC=O (compound **4**; λ_max_, nm (ε/M^−1^ cm^−1^): 211 (2682), 285 (br, 2340)), molybdenum precursor K_3_Na[MoO_2_(CN)_4_]·6H_2_O and the molybdenum complex **5** (λ_max_, nm (ε = M^−1^ cm^−1^): 225 (10653), 256 (sh, 2563), 265 (2758), 277 (2340), 323 (1023)) in acetonitrile solution.

Electronic spectra of the molybdenum precursor, ligand precursor **4** and the resulting Mo^IV^O complex (**5**) were recorded in CH_3_CN solution (Figure 3). The UV-vis spectra display absorption bands in the region 256–323 nm, characteristic of ligand to metal charge transfer (LMCT) and of intra-ligand charge transfer as also strongly suggested by comparison with the spectrum of the protected ligand. The two broad bands of rather similar shape for the ligand (ca. 250–310 nm) and complex **5** at λ_max_ 323 nm (ε = 1,023 M^−1^ cm^−1^) are most likely due to the same transition albeit shifted as would be expected after coordination. The corresponding bands and extinction coefficients of molybdenum complexes bearing two different dithiolene ligands, representing another type of non-symmetry, such as (Et_4_N)_2_[Mo^IV^O(S_2_C_2_(CO_2_Me)_2_)(bdtCl_2_)] (λ_max_ 531 nm; ε = 340 M^−1^ cm^−1^) (Sugimoto et al., 2009) and (Ph_4_P)_2_[Mo^IV^O(edt)(mnt)] (λ_max_ 433 nm; ε = 1,110 M^−1^ cm^−1^) (Donahue et al., 1998) are comparable to those of complex **5** reported here. The intensities (extinction coefficients) of the reported bands are well in accordance, while the observed band energy for complex 5 is higher (band at lower wavelength). In the complex we therefore tentatively assign the band at 323 nm to an LLCT transition. The single very broad absorption signal belonging to the dithiolene ligand precursor **4** is narrowed and exhibits a bathochromic shift in complex **5** indicative of a significant change in the electronic structure with more distinct LLCT transitions at slightly lower energy, as expected upon loss of the protecting C=O function (potentially engaged in resonance structures), coordination to a metal center and formation of new mixed metal-ligand molecular orbitals. Similar observations were made previously with a series of moderately related strictly aliphatic complexes (cyclohexane, pyrane, and thiopyrane derived ligands) with signals in the region 260–476 nm (Sugimoto et al., 2005). Coucouvanis and co-workers reported UV-vis data of the complex (Et_4_N)_2_[MoO(S_2_C_2_COOMe)_2_], which can be considered the closest relative of the new complex at λ_max_: 360, 460(sh) and 550 nm (Coucouvanis et al., 1991) albeit without extinction coefficients. Replacing the strongly electron withdrawing ester function by the ethyl alcohol substituent apparently shifts the transitions to higher energy; possibly also due to increasing ligand field strength when more electron density can be pushed toward the central metal. The recorded values for **5** are further comparable to related (Et_4_N)_2_[MoO(S_2_C_2_(COPh)_2_)_2_] with phenyl-keto substituents (λ_ma_x: 310 (sh), 338 (sh), 400 nm), the bands of which fall in between those of complex **5** and the Coucouvanis complex (Ansari et al., 1987).

The observed shift in the UV-vis data from the Cocouvanis complex (all ester) to complex **5** (mixed; ester and alcohol) corresponds also to the electrochemical properties of both. The cyclic voltammogram of **5** (see Figure S26) exhibits a reversible redox process for the Mo^IV^↔Mo^V^ transition ([MoO(mohdt)_2_]^2−^/[MoO(mohdt)_2_]^−^) at −0.62 V *vs*. [Fc]/[Fc]^+^ which constitutes a decrease in potential compared to the all ester complex at −0.074 V *vs*. [Fc]/[Fc]^+^ (value given in the report: −0.03 V vs. SCE) (Coucouvanis et al., 1991); i.e., the electron pushing alcohol substituent facilitates oxidation of the complex whereas the complex with two electron withdrawing ester substituents is more easily reduced.

### Structural Characterization

**Figure 4:**
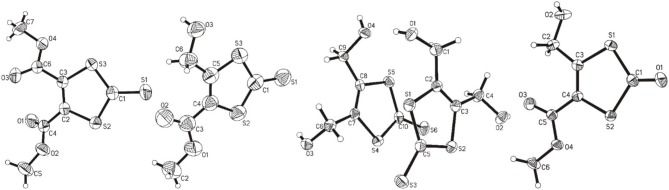
From left to right: the molecular structures of **1**, **2**, **3**, and **4**, respectively, shown at the 50% probability level.

The molecular structures of **1**, **2**, **3**, and **4** are shown in Figure 4 and the selected comparable bond distances and angles are listed in Table 2. All ligands were (re-)crystallized by the slow diffusion method. The structure of **1** was published previously in a databank without any accompanying discussion (Neil Bricklebank et al., 2003). In the X-ray structure of compound **3** two independent molecules are present in the unit cell, which differ slightly with respect to the angles of the -CH_2_-OH substituents to the ene moiety (rmsd 0.305; max. distance 0.5464 Å). All three ene-trithiocarbonate compounds (**1**–**3)** crystallized in the monoclinic *P*2_1_/*c* space group whereas compound **4** (ene-dithiocarbonate) crystallized in the monoclinic *P*2_1_/*n* space group. A description of a crystal structure of **3** in a different space group (*C*2/*c*) and with only one molecule in the asymmetric unit is available in *Acta Cryst* (Pløger et al., 2006). The ene-trithiocarbonate rings are structurally all similar and exhibit C=S, C–S and C=C distances in ranges of 1.632(4)−1.659(3), 1.718(3)−1.748(3) and 1.339(4)−1.347(4) Å, respectively.

**Table 2:** Selected bond lengths [Å] and angles [°] for **1**, **2, 3**, and **4**.

	**1**	**2**	**3**	**3**	**4**
			**Trithiocarbonate mole 1**	**Trithiocarbonate mole 2**	
**LENGTHS**					
C=S/O	1.636(3)	1.632(4)	1.659(3)	1.641(3)	1.204(2)
C–S (A)	1.727(3)	1.718(4)	1.718(3)	1.728(3)	1.776(2)
C–S (B)	1.732(3)	1.740(4)	1.659(3)	1.728(3)	1.760(2)
C=C	1.345(4)	1.346(5)	1.339(4)	1.347(4)	1.349(3)
**ANGLES**					
S=C–S (A)	122.50(18)	124.3(2)	123.85(17)	123.1(2)	123.17(16)
S=C–S (B)	124.59(18)	122.9(2)	122.78(19)	124.71(19)	123.79(16)
S–C–S	112.90(16)	112.8(2)	113.36(17)	112.18(16)	113.03(11)
C=C–S (A)	116.3(2)	115.9(3)	116.4(2)	115.7(2)	116.75(15)
C=C–S (B)	116.5(2)	116.5(3)	116.2(2)	116.0(2)	118.10(15)

**Figure 5:**
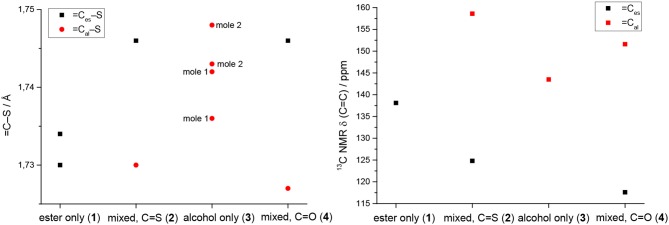
Ene-carbon to sulfur (=C–S) distances in Å (left) and ^13^C NMR chemical shifts of the ene moiety in ppm (right) visualizing the substituents' influence in compound **1** (ester only), compound **2** (mixed ester/alcohol), compound **3** (alcohol only; the individual molecules of the structure are indicated by mole 1 and mole 2 in the graph) and compound **4** (mixed ester/alcohol) strongly supporting the proposed push-pull effect in the asymmetric molecules. =C_es_-S and =C_al_-S indicate the ene-carbon atoms which are substituted by the ester and alcohol functions, respectively.

However, the ene carbon atoms' C–S bond distances in the four ligand precursor molecules upon close inspection are rather noteworthy. The intention of utilizing distinct substituents and an asymmetrically substituted dithiolene ligand for the complex synthesis was to fine-tune the electronic properties of the ligand (and consequently of the complex) hypothesizing that with a push-pull-effect the ligand's non-innocence (i.e., its ability to donate electron density/electrons to the metal center) should be raised. While one half of the ene-dithiolate moiety has a stronger preference for donating electron density toward the metal center than the other, then such donation should be facilitated compared to a system in which both substituents compete for the exact same effect and having the exact same properties. The resonance of such system, however, is expected to be decreased, translating into lower stability which is typically concomitant to higher reactivity (secondary effect; see Figure S27). That such primary effect was indeed realized at least in the ligand precursors is strongly supported by the distances between the ene-carbon atoms and the sulfur atoms as well as by the ^13^C-NMR data as already discussed above (Figure 5). The two (or four in case of **3** with the two independent molecules in the asymmetric unit) ene-C-S bond distances in the symmetric molecules of **1** and **3** are much more similar to each other than to those in the unsymmetric molecules of **2** and **4**. Most notably, the C-S bond lengths involving the ester substituted ene carbon are significantly longer in **2** and **4** than in the case of **1** (ester only) and those involving the alcohol substituted ene carbon atoms are much shorter than in the case of **3** (alcohol only). The unsymmetric substitution apparently increases the C-S single bond character of the ester side of the ene-dithio moiety and the C-S double bond character of the alcohol side. The latter will facilitate electron density donation toward the coordinated metal upon complex formation from this side of the molecule, as there is apparently already more density available in the respective bonds compared to the ester sides of the molecules. These metrical observations coincide with ^13^C-NMR data of the ene functional group discussed above. In fact, the chemical shifts of the symmetric molecules (**1** and **3**) are even closer to each other than they are to the shifts of either of the ene carbon atoms in the unsymmetric molecules. This means, that exchanging just one of two substituents results in a stronger modulation of the electronic structure compared to replacing both substituents.

With respect to the influence of the protecting group, the C=O oxo distance in **4** is 1.204(2) Å, which is necessarily shorter than the C=S distances in ene-trithiocarbonates due to the smaller size of oxygen atoms compared to sulfur. The other bond distances of the ene-dithiocarbonate moiety are slightly longer than the observed ranges for the ene-tritiocarbonates (OC–S: 1.760/1.776 Å; C=C 1.349 Å) (see Table 2). This indicates somehow stronger donation of electron density toward the C=O functional group of the ene-dithiocarbonate than to the respective C=S of the ene-trithiocarbonates.

**Figure 6:**
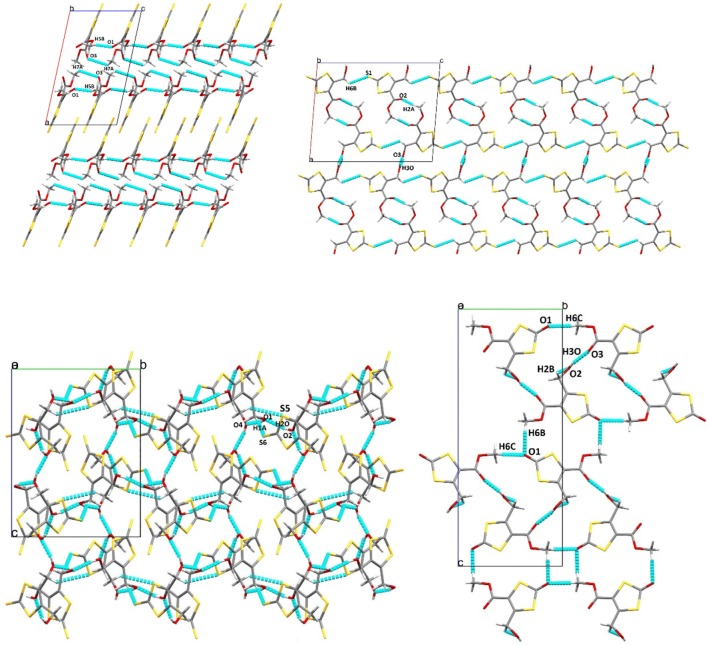
Crystal packing projection views along the *b* axis of **1** (top left), and **2** (top right), and along the *a* axis of **3** (bottom left) and **4** (bottom right). Selected hydrogen bonding/short contacts (Å): (**1**): C(5)–H(5B)···O(1): 2.522, C(7)–H(7A) ···O(3): 2.708, C(4)–O(3): 3.001; (**2**): O(3)–H(3O)···O(3): 1.89(6), C(2)–H(2A)···O(2): 2.59, S(1)–H(6B): 2.960; (**3**): O(1)–H(1O)···O(4): 1.95(4), CO(2)–H(2O)···O(1): 1.90(4), O(3)–H(3O)···O(2): 2.10(4), O(4)–H(4O)···O(3): 1.89(4), H(1A)–S(6): 2.946, S(5)–H(9B): 2.979; (**4**): O(1)–H(6C): 2.503, O(2)–H(2B): 2.441, O(3)–H(3O): 1.999, O(1)–H(6B): 2.594.

Figure 6 shows projections of the crystal packing in the structures of **1**, **2**, **3**, and **4** along the *a* or *b* axes. The hydrogen bonding/short contacts present in all structures are depicted in blue. Only for **1** all ene-trithiocarbonate moieties are coplanar within the crystal lattice whereas for the other three compounds the planar parts of the molecular structures are arranged in angles up to nearly perpendicular (87.63°; **3**) to each other. X-ray suitable single crystals of complex **5** remained elusive, unfortunately, despite considerable and repeated efforts of recrystallization.

### OAT Catalysis

**Scheme 3:**
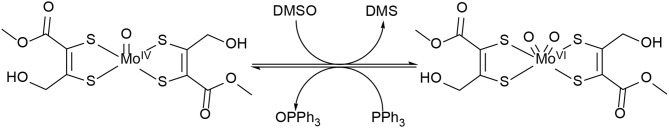
Proposed reactions for the oxygen atom transfer catalyzed by **5**.

The OAT activity of Mo^IV^O bis-dithiolene complex **5** was investigated with the model oxygen atom transfer reaction between DMSO and PPh_3_ (see Scheme 3; based on Ref. Berg and Holm, 1985). The reaction progress was monitored by ^31^P-NMR spectroscopy. The reaction typically proceeds *via* oxygen atom transfer from DMSO to the Mo^IV^O moiety resulting in dimethyl sulfide and a Mo^VI^O_2_ species which then oxidizes the acceptor substrate PPh_3_ yielding OPPh_3_ and concurrently completing the catalytic cycle (Lorber et al., 1997; Tucci et al., 1998). In this mechanism, the phosphorous atom of the alkyl-phosphines is performing a nucleophilic attack on one of the two oxido ligands on the Mo^VI^ center by donation into the empty Mo=O π^*^ orbital generating the phosphine oxide intermediate, while a free electron pair of the oxygen atom is simultaneously attacking the P–C σ^*^ orbital (Holm, 1987; Smith et al., 2000). I.e., an electron pair on phosphorous establishes an initial single bond with oxygen and for the respective P=O double bond a lone pair on oxygen is used. At the same time one electron pair of the Mo=O double bond becomes the new second lone pair on oxygen and the other electron pair of the former metal oxygen double bond remains entirely at the metal center (severing the bond between Mo and O), so that the two-electron reduction of the metal/oxidation of phosphorous proceeds smoothly together with the transfer of oxygen from metal to substrate.

**Figure 7:**
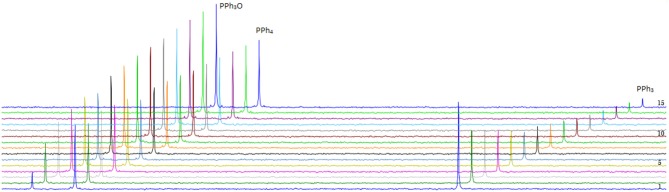
Transformation of PPh_3_ to OPPh_3_ catalyzed by **5** in the presence of PPh_3_ and DMSO-d6 as oxidizing agent monitored by ^31^P-NMR spectroscopy at r.t (unregulated temperature). Fifteen spectra are shown taken with a frequency of 1 every 4 h.

DMSO is used as oxygen donor source and simultaneously employed as solvent and substrate with consequentially very high excess to the catalyst. PPh_3_ was chosen as expedient model substrate for its high solubility in organic solvents and its suitable affinity toward oxygen. A 3 mM catalyst loading was employed together with 3 eq. of PPh_3_ and 0.5 mL of deoxygenated DMSO in an airtight NMR tube at room temperature. ^31^P-NMR spectroscopy is the most convenient method to monitor the reaction progress since substrate (PPh_3_) and product (PPh_3_O) demonstrate well-separated resonance signals (PPh_3_: s, −5.8 ppm and PPh_3_O: s, 26.6 ppm in DMSO-*d*_6_). Reaction monitoring by NMR started immediately after preparation of the reaction mixture under N_2_ atmosphere. The concentration of PPh_3_ (at −5.8 ppm) decreased gradually with the reaction time and at the end of the reaction PPh_3_O was the dominating species (Figure 7).

As the central metal is involved in a two electron redox-process, electron density buffering by a non-innocent ligand is considered beneficial for such reactions. The aim of this study was to optimize this electron density buffering by the asymmetric ligand substitution and a respective push-pull effect (present in the ligand precursor as evidenced by structural and spectroscopic data). A one-sided preference for electron donation induced by the introduction of one electron donating alcohol substituent was supposed to better support the involved redox processes and increase the complex' reactivity. However, the reaction proceeds very slowly with the maximum conversion (93%) of applied PPh_3_ (9 mM) reached after ~2.5 days with a not entirely steady progress under the applied reaction conditions (rather hot summer days, cooler nights, no temperature control; see Figure S22). We therefore abstained from trying to extract specific kinetic parameters for this transformation.

The disappointingly low reaction velocity can be attributed not entirely to the low activity of **5** but also to the comparably mild oxidizing substrate. Although well-established by the Holm group, the DMSO/PPh_3_ system has its disadvantages with respect to the known very slow conversion of Mo^IV^ to Mo^VI^ with DMSO. When adding Me_3_NO as a stronger oxidizing agent to a freshly prepared solution of **5** and PPh_3_ in DMSO-d6 we observed 36.52% conversion overnight (within 15 h), which constitutes a slight acceleration in comparison but still not the anticipated rapid catalytic process.

The most frequently investigated molybdenum centers in oxidoreductase model chemistry are Mo^IV^O and Mo^VI^O_2_ species which are comparable to the native co-factors regarding the oxidation states and they bear transferable oxido ligands (McMaster et al., 2004b). These complexes, however, in particular when mixed in a reaction medium while circling through catalysis, can also form dimeric or oligomeric assemblies transforming terminal oxido ligands into μ-oxido functions, e.g., dimeric and chemically inert M${\text{o}}_{2}^{\text{V}}$O_3_ moieties, which are catalytically inactive (McMaster et al., 2004a; Mitra and Sarkar, 2013; Hille et al., 2016). Confirming their chemical structures, a number of X-ray diffraction studies of such dimeric species are reported in the literature (Tatsumisago et al., 1982; Ratnani et al., 1990; Sellmann et al., 1992; Thompson et al., 1993; Awwal et al., 2007; Pal et al., 2007; Mitra and Sarkar, 2013) albeit not with bis-dithiolene molybdenum centers. In fact Subramanian et al. have stated that the reduction of Mo^VI^O_2_L_2_ species necessarily leads to μ-oxo-bridged dimers (Subramanian et al., 1984). The formation of such species constitutes a general problem associated with catalytic/kinetic investigations of OAT, although in the best cases monomeric Mo^IV^O plus Mo^VI^O species and dimeric M${\text{o}}_{2}^{\text{V}}$O_3_ are in equilibrium with considerable amounts left of the former pair so that catalytic activity can still persist (Holm, 1987, 1990).

In order to verify whether the slowness of the OAT reaction was indeed due to dimer formation, a solution of 3 mM complex **5** in the presence of oxidizing agent trimethylaminoxid (TMAO) in acetonitrile was prepared and monitored by UV-Vis spectroscopy under anaerobic condition. It was observed that after the initial formation of the transient Mo^VI^O_2_ species (here very broad signal at λ_max_: 540 nm), it swiftly decomposed again while the signal for the catalytically inert M${\text{o}}_{2}^{\text{V}}$O_3_ species (typical signal at λ_max_: 375 nm) (Villata et al., 2000; Sugimoto et al., 2003; Pal et al., 2007) exhibited a steady rise (see Figure S22). The change in the UV-vis with the progress of the reaction exhibits clean isosbestic points indicating the simultaneous presence of only two (not three) species. This can be explained by the fact that the transient di-oxo species is of particularly low concentration, hence, nearly invisible in the UV-vis as the dimer formation is essentially instant as soon as the di-oxo species is available.

The typical dimerization is particularly problematic for those systems with bis-dithiolene co-ligands bearing aliphatic backbones without steric or electronic protection. When aromatic dithiolene ligands are used (and this refers to both, an aromatic substituent on the ene- moiety as well as the ene moiety being part of an aromatic ring as in benzene-dithiolate) molybdenum dithiolene complexes are typically much more stable and, hence, much less reactive than those with aliphatic dithiolene systems (Fischer and Fischer, 2017). Dithiolene ligands bearing electron withdrawing groups as substituents typically exhibit weak Mo–S bonds. This does promote the Mo=O bond and concomitantly stabilizes the monomeric species by electronic tuning but it also decreases the catalytic activity (Hille et al., 2016). In contrast, dithiolene ligands with electron donating groups push electron density toward the metal center and by that decrease the Lewis acidity of molybdenum. It was shown previously that the Mo=O bond is weakened in dithiolene complexes with aliphatic backbones in particular with electron donating substituents (Hille et al., 2016). Taking all this into consideration we attribute the slowness of the catalyzed OAT reaction observed for complex **5** predominantly to the formation of the dimeric and chemically inert M${\text{o}}_{2}^{\text{V}}$O_3_ species as the aliphatic substituents on the used dithiolene ligand have mixed electron donating and electron withdrawing character, intended to fine tune the electronic structure of the complex and balance stability and reactivity. The dimerization may benefit from the presence of ester and hydroxyl functions on the dithiolene which constitute excellent functionalities for hydrogen bonding, which potentially results in close proximity of the catalytic centers even in solution. Previously, the presence of hydrogen bonding potential, in particular for intramolecular hydrogen bonding was perceived to be beneficial for catalysis (Oku et al., 1997; Okamura et al., 2016a,b). However, in this case it appears to be rather detrimental. The respective potential interactions between two complexes might facilitate formation of the catalytically inactive dimer by intermolecular hydrogen bonds (see Figure S23 for a proposed interaction). An accelerated dimer formation would slow down the catalytic OAT reaction significantly by inactivation of the present catalyst species and in addition by preventing the catalytically active species to diffuse freely into the solution. The observed actual reactivity, in fact, is more comparable to a system with aromatic backbone, e.g., to one previously reported by our group ((Bu_4_N)_2_[Mo^IV^O(ntdt)_2_] and (Ph_4_P)_2_[Mo^VI^O_2_(ntdt)_2_]; ntdt = 2-naphthyl-1,4-dithiolate) (Ghosh et al., 2017) than to other aliphatic systems. Although the strategy of combining electron pushing and electron withdrawing substituents generally appears to achieve the anticipated fine-tuning of the electronic structure (evident at least for the ligand precursor) it did not translate into the targeted increase in reactivity. Apparently, further consideration needs to go into the exact nature of the utilized substituents. For respective next generation compounds the ability to engage in hydrogen bonding should be assessed from the very beginning.

## Conclusions

The aliphatic dithiolene ligand, 1-methoxy-1-oxo-4-hydroxy-but-2-ene-2,3-bis-thiolate (mohdt) and its Mo bis-dithiolene complex were synthetized and comprehensively characterized. The unsymmetrically substituted dithiolene ligand is subject to a push-pull effect modulating its electronic structure. A comparison of structural-metrical and ^13^C-NMR data of four related ligand precursor compounds reveals that substituent effects are indeed much more pronounced in unsymmetric than in symmetric molecules. ^13^C-NMR data in particular turned out to be rather sensitive to such effects and should be considered a valuable tool for respective assessments. Since the synthesized complex can be considered a structural model for the molybdopterin bearing DMSO reductases with respect to the immediate coordination sphere, it was also tested for its catalytic oxygen atom transfer ability in DMSO by mixing the catalyst with PPh_3_ at room temperature. The molybdenum complex catalyzes the OAT reaction from DMSO to PPh_3_ up to a 93% conversion within 56 h. In contrast to the expectations based on the evidenced push-pull effect, the catalytic performance of complex **5** is unexpectedly slow most likely due to the formation of dimeric Mo^V^ species after initial oxidative transformation of Mo^IV^O to Mo^VI^O_2_ species. This is possibly supported by hydrogen bonding effects of the ligands' substituents and certainly not hindered by any steric bulk.

## Author Contributions

MA: syntheses, experiments, and drafting the manuscript. AG: syntheses, experiments, and supporting manuscript drafting. CF: scientific support of the project, catalysis, and kinetic evaluation. CS: study design, in charge of overall direction, managing the project, and finalizing the report.

### Conflict of Interest Statement

The authors declare that the research was conducted in the absence of any commercial or financial relationships that could be construed as a potential conflict of interest.
