# Supplementary material for: An Asymmetrically Substituted Aliphatic Bis-Dithiolene Mono-Oxido Molybdenum(IV) Complex With Ester and Alcohol Functions as Structural and Functional Active Site Model of Molybdoenzymes
Source: Front Chem. 2019 Jul 11;7:486. doi: 10.3389/fchem.2019.00486 (PMC6637267; doi:10.3389/fchem.2019.00486)
Supplement: Supplementary file 1 [file Data_Sheet_1.pdf]

Supplementary Material:

An Asymmetrically Substituted Aliphatic Bis-Dithiolene Mono-Oxido Molybdenum(IV) Complex with Ester and Alcohol Functions as Structural and Functional Active Site Model of Molybdoenzymes

Mohsen Ahmadi[1], Christian Fischer[1], Ashta Chandra Ghosh[2], Carola Schulzke[1]*

[1]Institut für Biochemie, Universität Greifswald, Felix-Hausdorff-Str. 4, 17487 Greifswald, Germany

[2]Institute of Condensed Matter and Nanosciences, Molecules, Solids and Reactivity (IMCN/MOST), Université catholique de Louvain, Place L. Pasteur 1, 1348 Louvain-la-Neuve, Belgium

*** Correspondence:** Carola Schulzke: carola.schulzke@uni-greifswald.de

CONTENTS

Spectral Characterization 5

Figure S1. ^{1}H NMR of dimethyl 2-thioxo-1,3-dithiole-4,5-dicarboxylate (**1**) in $CDCl_3$ at 25 ^{o}C. 5

Figure S2. ^{13}C NMR of dimethyl 2-thioxo-1,3-dithiole-4,5-dicarboxylate (**1**) in $CDCl_3$ at 25 ^{o}C. 6

Figure S3. APCI mass spectrum of dimethyl 2-thioxo-1,3-dithiole-4,5-dicarboxylate (**1**) in MeOH. 6

Figure S4. FT-IR spectrum of dimethyl 2-thioxo-1,3-dithiole-4,5-dicarboxylate (**1**) in KBr pellet. 6

Figure S5. ^{1}H NMR of methyl 5-(hydroxymethyl)-2-thioxo-1,3-dithiole-4-carboxylate (**2**) in $CDCl_3$ at 25 ^{o}C. 7

Figure S6. ^{13}C NMR of methyl 5-(hydroxymethyl)-2-thioxo-1,3-dithiole-4-carboxylate (**2**) in $CDCl_3$ at 25 ^{o}C. 7

Figure S7. FT-IR spectrum of methyl 5-(hydroxymethyl)-2-thioxo-1,3-dithiole-4-carboxylate (**2**) in KBr pellet. 8

Figure S8. APCI mass spectrum of methyl 5-(hydroxymethyl)-2-thioxo-1,3-dithiole-4-carboxylate (**2**) in MeOH at 25 ^{o}C. 8

Figure S9. ^{1}H NMR of 4,5-bis(hydroxymethyl)-1,3-dithiole-2-thione (**3**) in MeOD at 25 ^{o}C. 9

Figure S10. ^{13}C NMR of 4,5-bis(hydroxymethyl)-1,3-dithiole-2-thione (**3**) in MeOD at 25 ^{o}C. 9

Figure S11. APCI mass spectrum of 4,5-bis(hydroxymethyl)-1,3-dithiole-2-thione (**3**) in MeOH at 25 ^{o}C. 10

Figure S12. FT-IR spectrum of 4,5-bis(hydroxymethyl)-1,3-dithiole-2-thione (**3**) in KBr pellet. 10

Figure S13. ^{1}H NMR of 4-methyl-carboxylate-5-hydroxymethyl-1,3-dithiole-2-one (**4**, *mohdtC=O*) in $CDCl_3$ at 25 ^{o}C. 11

Figure S14. ^{13}C NMR of 4-methyl-carboxylate-5-hydroxymethyl-1,3-dithiole-2-one (**4**, *mohdtC=O*) in $CDCl_3$ at 25 ^{o}C. 11

Figure S15. APCI mass spectrum of 4-methyl-carboxylate-5-hydroxymethyl-1,3-dithiole-2-one (**4**, *mohdtC=O*) in MeOH at 25 ^{o}C. 12

Figure S16. FT-IR spectrum of 4-methyl-Carboxylate-5-hydroxymethyl-1,3-dithiole-2-one (**4**, *mohdtC=O*) in KBr pellet. 12

Figure S17. ^{1}H NMR of $(PPh_4)_2[MoO(mohdt)_2]$ (**5**) in CD_3CN at 25 ^{o}C. 13

Figure S18. ^{13}C NMR of $(PPh_4)_2[MoO(mohdt)_2]$ (**5**) in CD_3CN at 25 ^{o}C. 13

Figure S19. (a) MALDI-TOF-MS spectrum of complex **5** obtained in negative ion linear mode using 2,5-dihydroxy benzoic acid (DHB) as matrix (thin layer technique). (b) ESI-MS (-) spectrum (high temperature/medium fragmentation) of the complex **5** in methanol. Insert: ESI-MS (+) spectrum of **5**. 14

Figure S20. FT-IR spectra of $(PPh_4)_2[MoO(mohdt)_2]$ (**5**) in KBr pellet at 25 ^{o}C. 15

Figure S21. Elemental analysis of complex **5**. Calc. (%): C, 60.62; H, 4.74; S, 11.16. 15

Figure S22. Increasing [PPh_3O] (mM) plotted against time (h) for the OAT reaction between DMSO and PPh_3 catalyzed by 5. Conditions: [PPh_3]0 = 9 mM; [catalyst] = 3 mM in DMSO (0.5 mL) at r.t.; concentration of PPh_3O was monitored by ^{31}P NMR spectroscopy every 4 hours (the times of day/night for each data point are indicated relating to the respective intensity of sunlight/temperature in the NMR-lab). The fitting procedure, which gave the best fit (cubic spline) was applied with routines as implemented in MatLab according to the book: A Practical Guide to Splines by Carl de Boor, Springer-Verlag New York, 1978. MatLab is using polynomials of degree 3 with special "not a knot" boundary conditions at both ends. In our special case we had 15 knots which we interpolated to 100 equidistant points to obtain a smooth line which is supposed to help the viewer to see the daytime dependence of the data points. The interpolation does by no means provide any kinetic information 16

Figure S23. Plausible hydrogen bonding interaction between monomers and dimer formation 17

Figure S24. Changes in the electronic spectrum of an acetonitrile solution of [$Mo^{IV}O(mohdt)_2]^{2-}$ (5) (0.3 mM) in the presence of trimethylaminoxid (TMAO). 17

Figure S25. UV-visible spectra of mohdt-CO **4** (top left) and **5** (top right) in acetonitrile in various concentration; Extinction coefficient diagram at corresponding λ_{max} (bottom). 18

Figure S26. A) Cyclic voltammogram of 1 mM solution of **5** (left) in CH_3CN containing 0.1 M of Bu_4NPF_6 as electrolyte (scan rate: 0.1 Vs^{-1}). B) Cyclic voltammogram of **5** at different scan rates for the reversible redox couple Mo^{IV}/Mo^{V} (right). The peak potentials were recorded vs. internal reference [Fc]/[Fc]$^+$ at 298 K. 19

Structural Characterization 19

Table S1. Atomic coordinates (x 10^4) and equivalent isotropic displacement parameters (A^2 x 10^3) for **1**. U(eq) is defined as one third of the trace of the orthogonalized Uij tensor 19

Table S2. Bond lengths [Å] and angles [°] for **1**. 20

Table S3. Anisotropic displacement parameters (A^2 x 10^3) for **1**. The anisotropic displacement factor exponent takes the form: -2 pi^2 [h^2 a*^2 U11 + ... + 2 h k a* b* U12] 21

Table S4. Hydrogen bonds for **1** [Å and °]. 22

Table S5. Atomic coordinates (x 10^4) and equivalent isotropic displacement parameters (A^2 x 10^3) for 2. U(eq) is defined as one third of the trace of the orthogonalized Uij tensor 22

Table S6. Bond lengths [Å] and angles [°] for **2**. 23

Table S7. Anisotropic displacement parameters (A^2 x 10^3) for **2**. The anisotropic displacement factor exponent takes the form: -2 pi^2 [h^2 a*^2 U11 + ... + 2 h k a* b* U12] 24

Table S8. Hydrogen coordinates (x 10^4) and isotropic displacement parameters (A^2 x 10^3) for **2**. 24

Table S9. Hydrogen bonds for **2** [Å and °]. 25

Table S10. Atomic coordinates (x 10^4) and equivalent isotropic displacement parameters (A^2 x 10^3) for **3**. U(eq) is defined as one third of the trace of the orthogonalized Uij tensor 25

Table S11. Bond lengths [Å] and angles [°] for **3**. 26

Table S13. Hydrogen coordinates (x 10^4) and isotropic displacement parameters (A^2 x 10^3) for **3**. 28

Table S14. Hydrogen bonds for **3** [A and deg.]. 29

Table S15. Atomic coordinates (x 10^4) and equivalent isotropic displacement parameters (A^2 x 10^3) for **4**. U(eq) is defined as one third of the trace of the orthogonalized Uij tensor........ 29

Table S16. Bond lengths [Å] and angles [°] for **4**........ 30

Table S17. Anisotropic displacement parameters (A^2 x 10^3) for **4**. The anisotropic displacement factor exponent takes the form: -2 pi^2 [h^2 a*^2 U11 + ... + 2 h k a* b* U12]........ 31

Table S18. Hydrogen coordinates (x 10^4) and isotropic displacement parameters (A^2 x 10^3) for **4**. 32

Proposed Resonance Structures of 4 (Figure S27)........ 32

Spectral Characterization

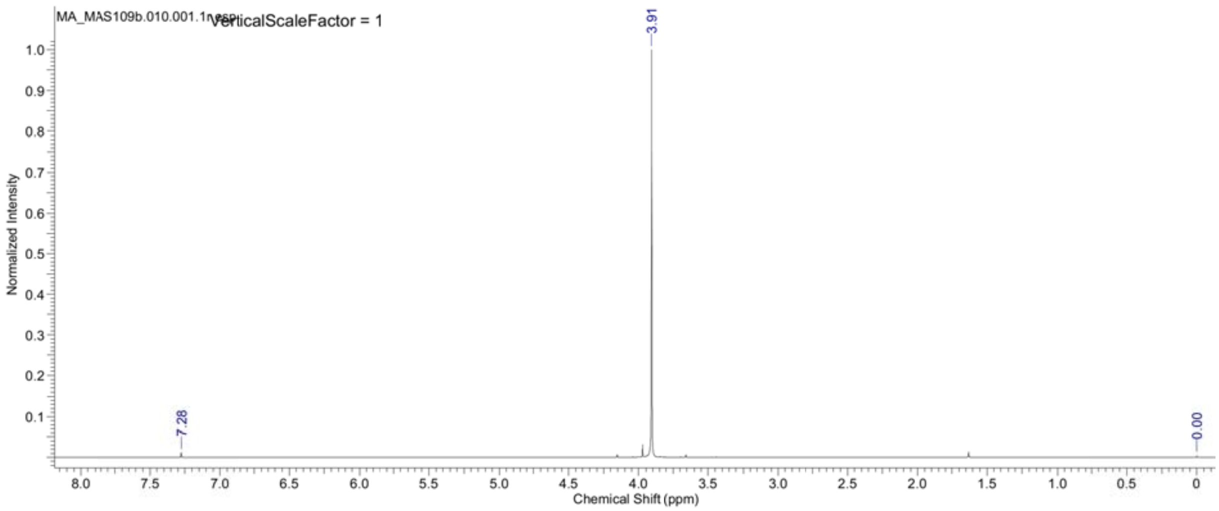

Figure S1. ^{1}H NMR of dimethyl 2-thioxo-1,3-dithiole-4,5-dicarboxylate (**1**) in $CDCl_3$ at 25 ^{o}C.

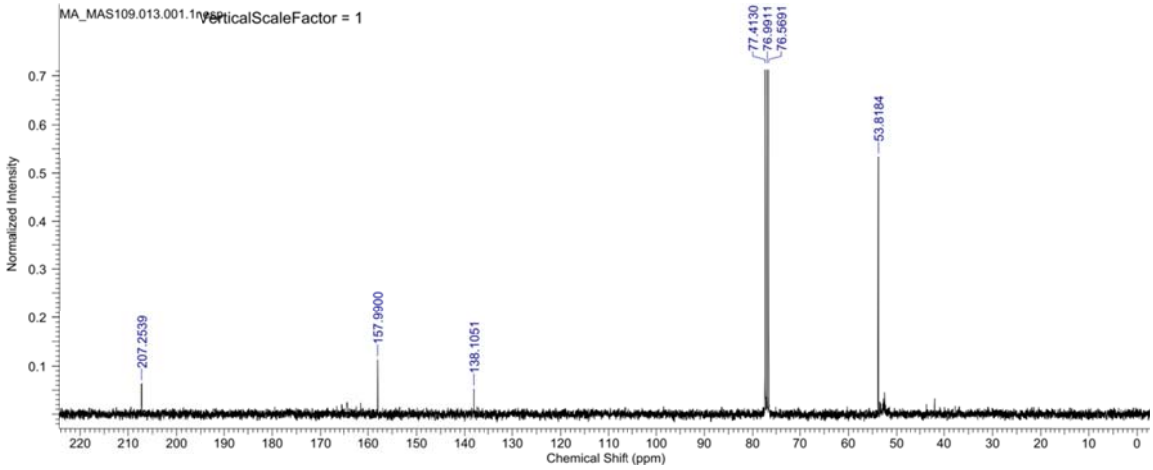

Figure S2. ^{13}C NMR of dimethyl 2-thioxo-1,3-dithiole-4,5-dicarboxylate (**1**) in $CDCl_3$ at 25 ^oC.

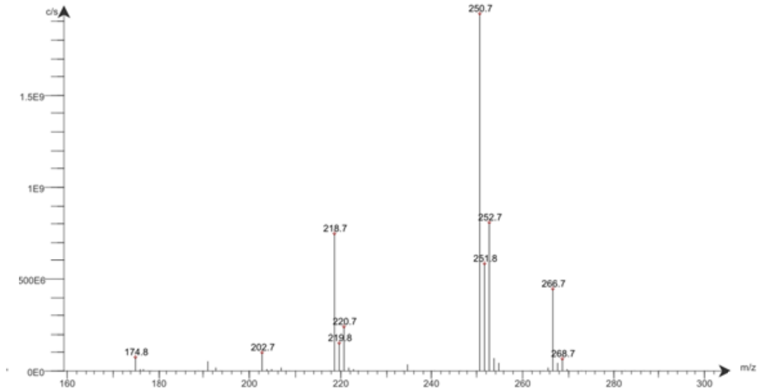

Figure S3. APCI mass spectrum of dimethyl 2-thioxo-1,3-dithiole-4,5-dicarboxylate (**1**) in MeOH.

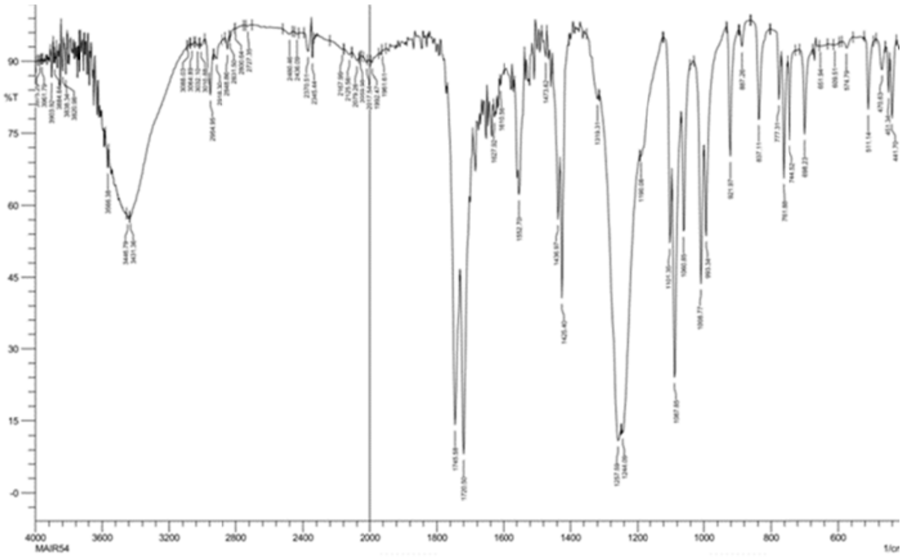

Figure S4. FT-IR spectrum of dimethyl 2-thioxo-1,3-dithiole-4,5-dicarboxylate (**1**) in KBr pellet.

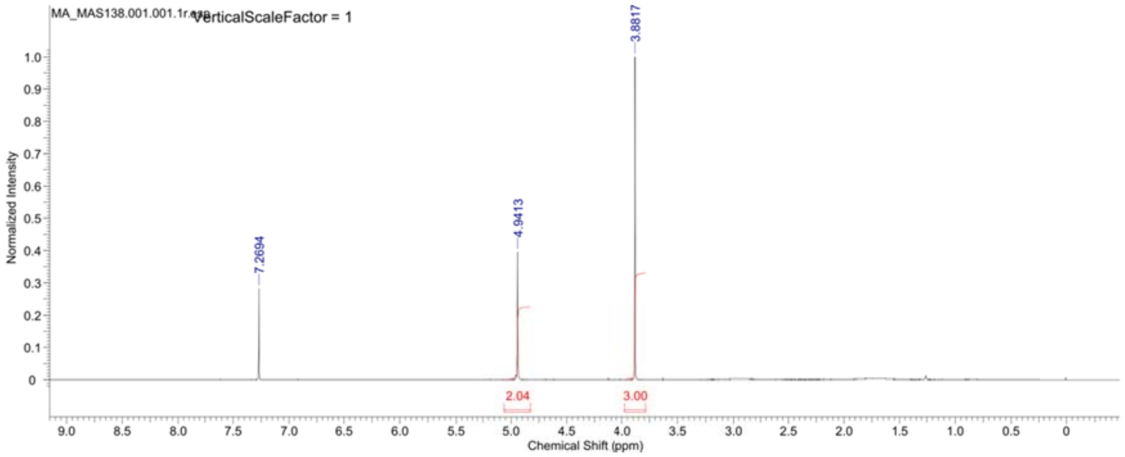

Figure S5. ^{1}H NMR of methyl 5-(hydroxymethyl)-2-thioxo-1,3-dithiole-4-carboxylate (**2**) in $CDCl_3$ at 25 ^{o}C.

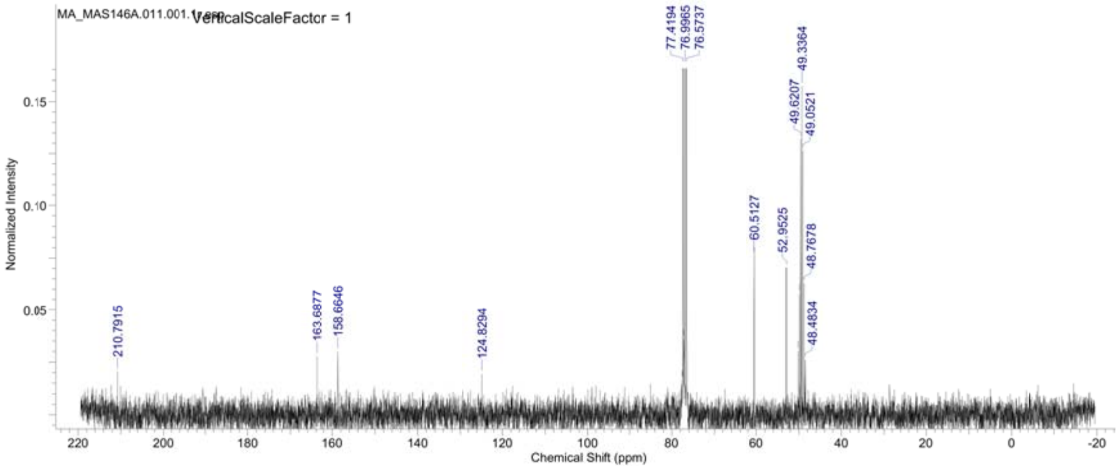

Figure S6. ^{13}C NMR of methyl 5-(hydroxymethyl)-2-thioxo-1,3-dithiole-4-carboxylate (**2**) in $CDCl_3$ at 25 ^{o}C.

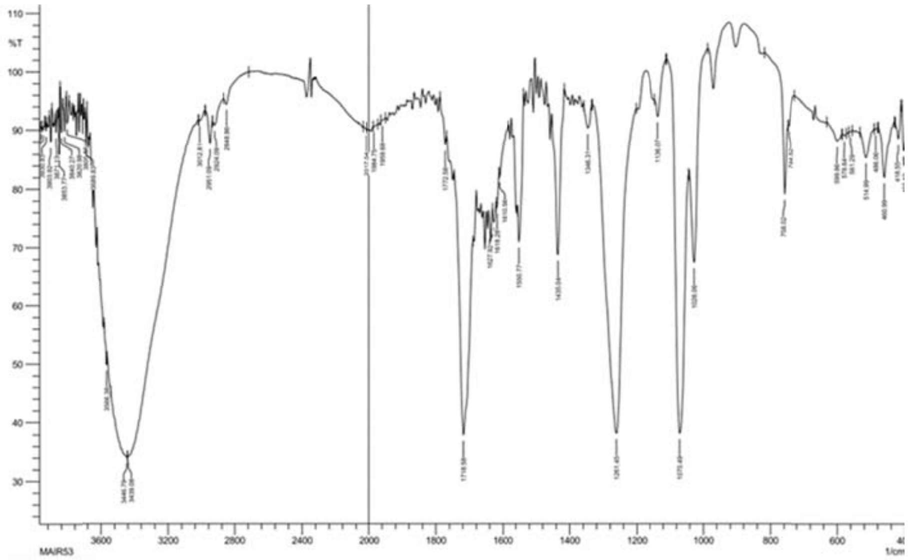

Figure S7. FT-IR spectrum of methyl 5-(hydroxymethyl)-2-thioxo-1,3-dithiole-4-carboxylate (**2**) in KBr pellet.

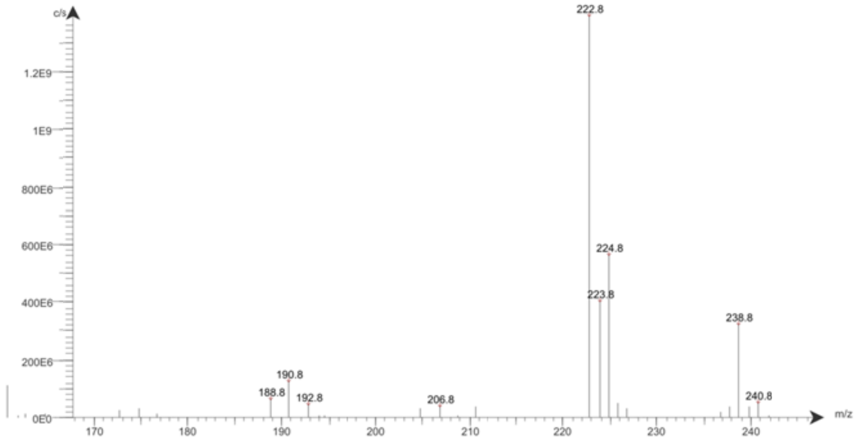

Figure S8. APCI mass spectrum of methyl 5-(hydroxymethyl)-2-thioxo-1,3-dithiole-4-carboxylate (**2**) in MeOH at 25 °C.

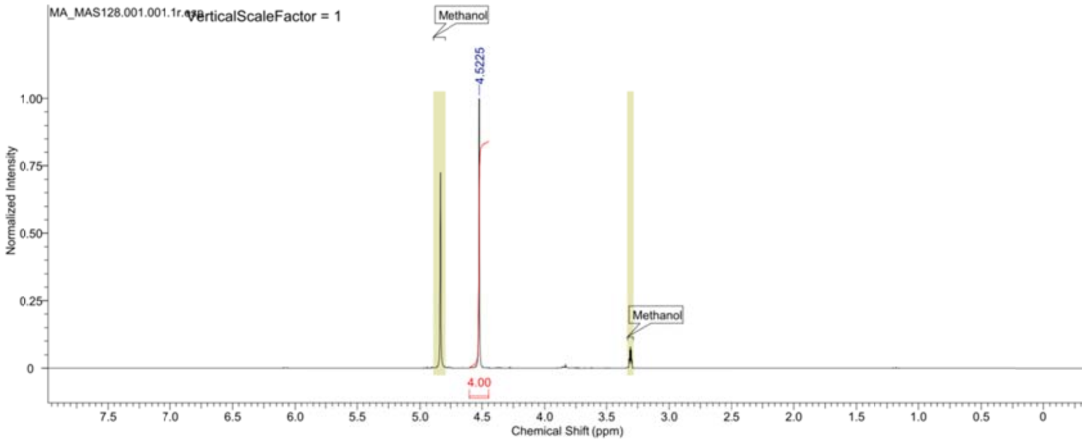

Figure S9. ^{1}H NMR of 4,5-bis(hydroxymethyl)-1,3-dithiole-2-thione (**3**) in MeOD at 25 °C.

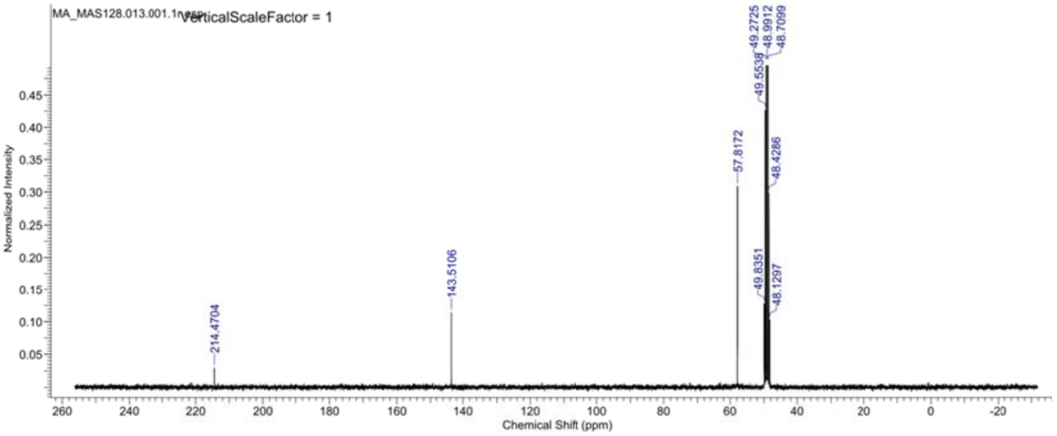

Figure S10. ^{13}C NMR of 4,5-bis(hydroxymethyl)-1,3-dithiole-2-thione (**3**) in MeOD at 25 °C.

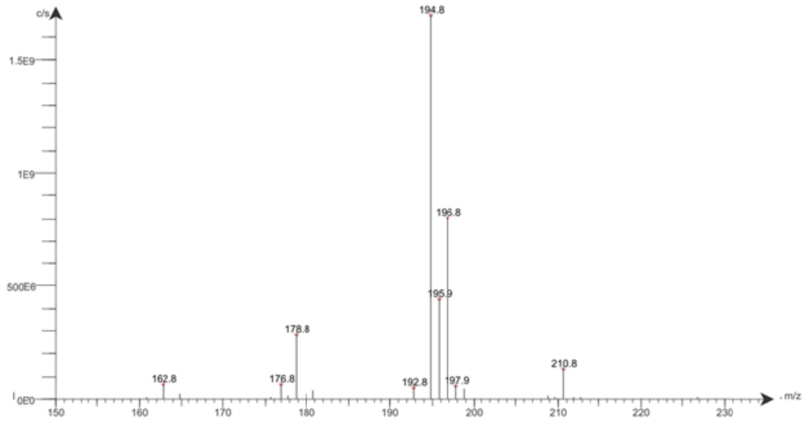

Figure S11. APCI mass spectrum of 4,5-bis(hydroxymethyl)-1,3-dithiole-2-thione (**3**) in MeOH at 25 ^{o}C.

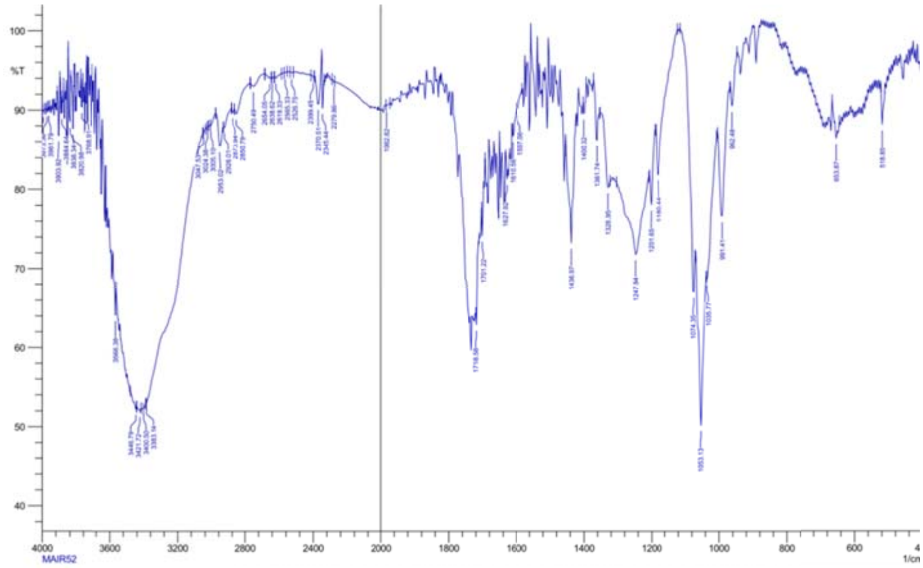

Figure S12. FT-IR spectrum of 4,5-bis(hydroxymethyl)-1,3-dithiole-2-thione (**3**) in KBr pellet.

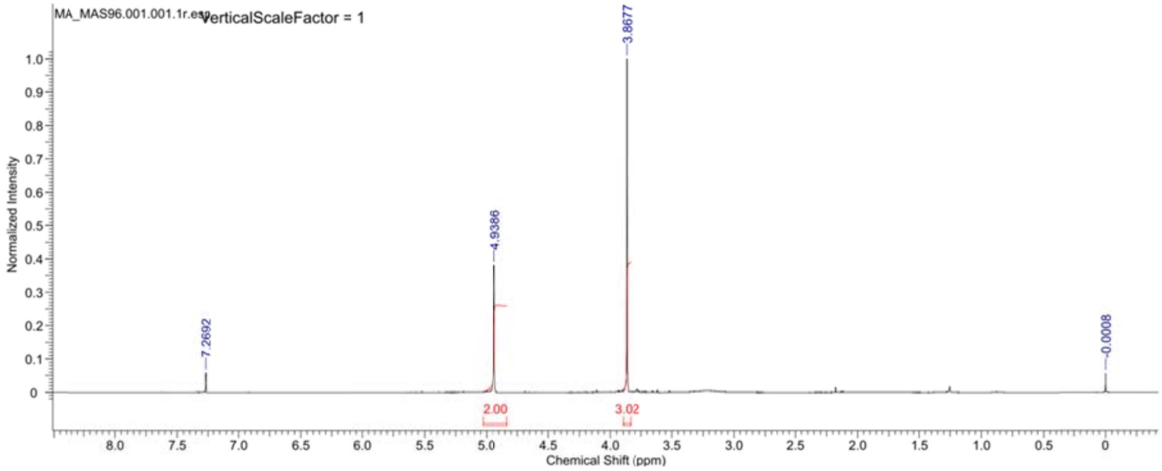

Figure S13. ^{1}H NMR of 4-methyl-carboxylate-5-hydroxymethyl-1,3-dithiole-2-one (**4**, *mohdtC=O*) in $CDCl_3$ at 25 ^{o}C.

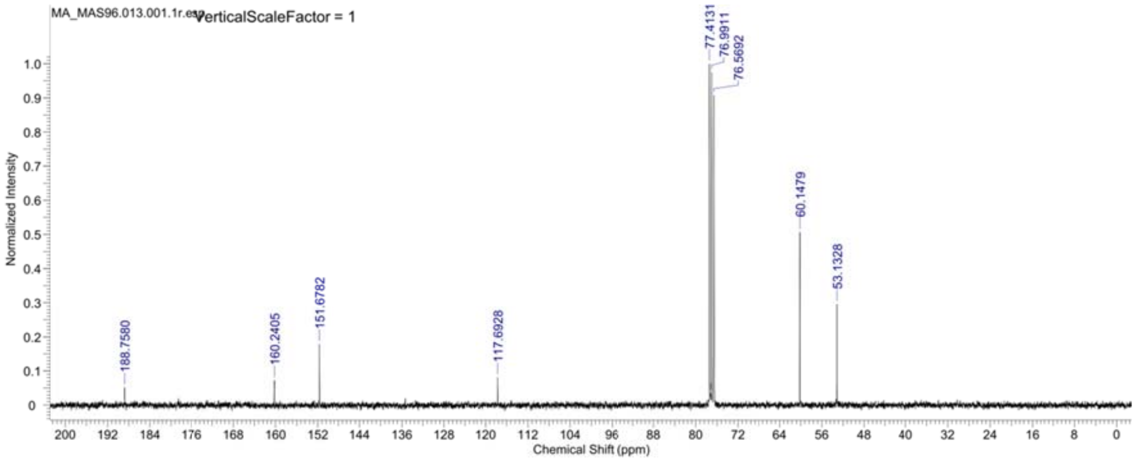

Figure S14. ^{13}C NMR of 4-methyl-carboxylate-5-hydroxymethyl-1,3-dithiole-2-one (**4**, *mohdtC=O*) in $CDCl_3$ at 25 ^{o}C.

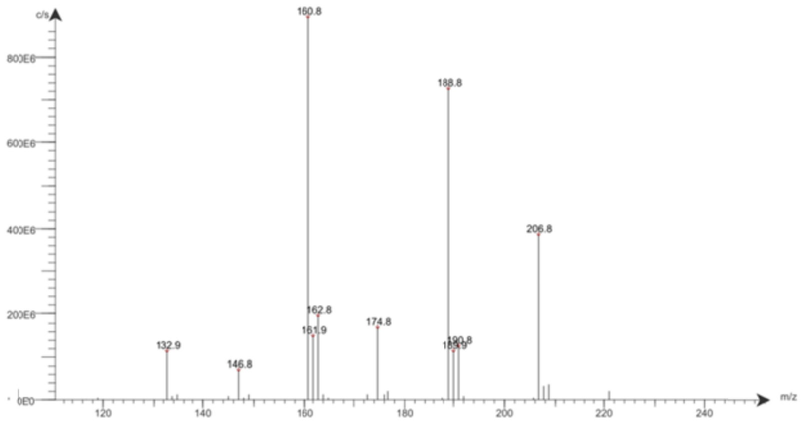

Figure S15. APCI mass spectrum of 4-methyl-carboxylate-5-hydroxymethyl-1,3-dithiole-2-one (**4**, *mohdtC=O*) in MeOH at 25 ^{o}C.

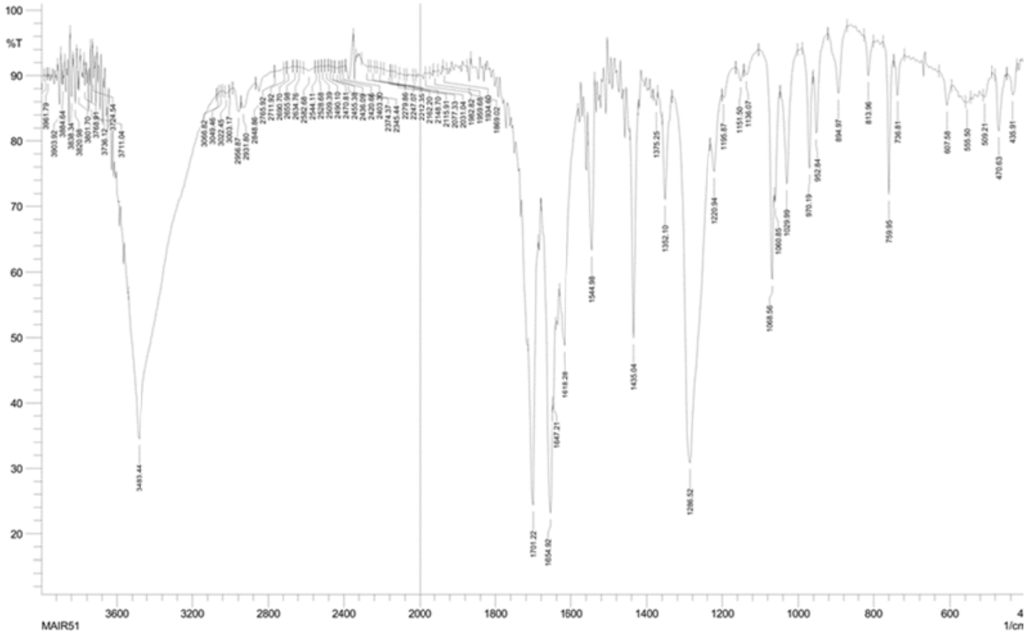

Figure S16. FT-IR spectrum of 4-methyl-Carboxylate-5-hydroxymethyl-1,3-dithiole-2-one (**4**, *mohdtC=O*) in KBr pellet.

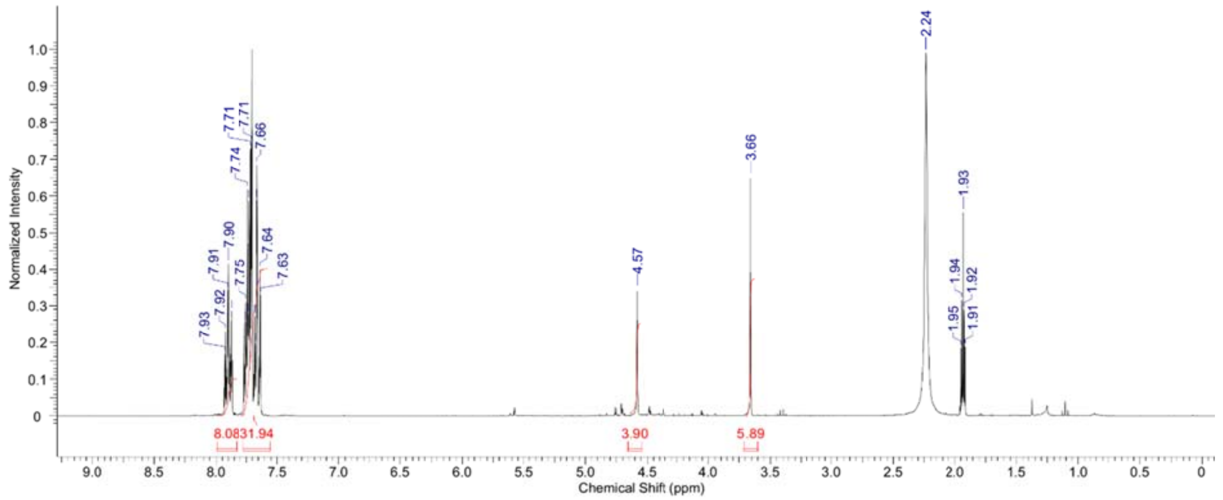

Figure S17. ^{1}H NMR of $(PPh_4)_2[MoO(mohdt)_2]$ (**5**) in CD_3CN at 25 °C.

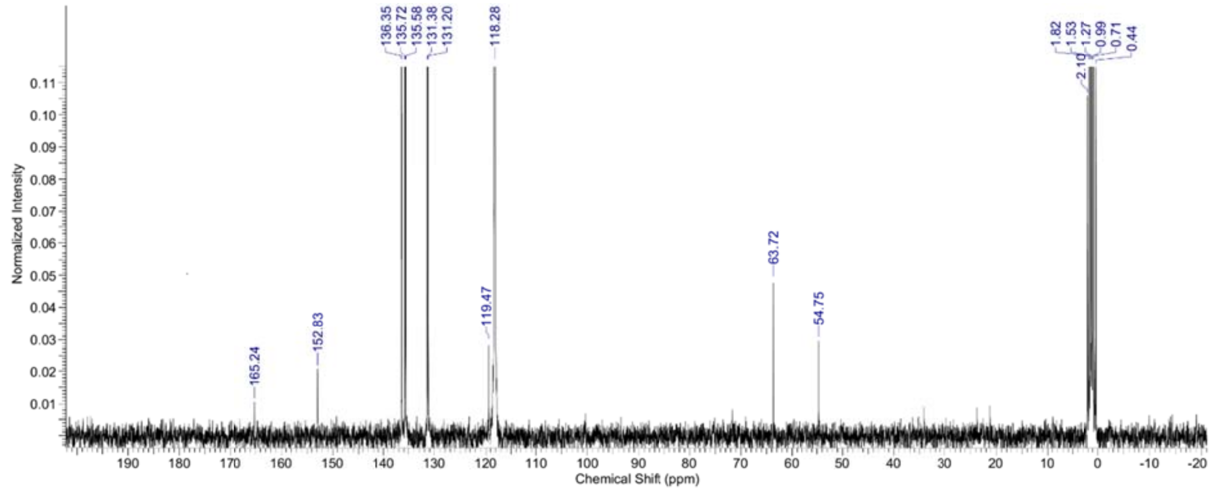

Figure S18. ^{13}C NMR of $(PPh_4)_2[MoO(mohdt)_2]$ (**5**) in CD_3CN at 25 °C.

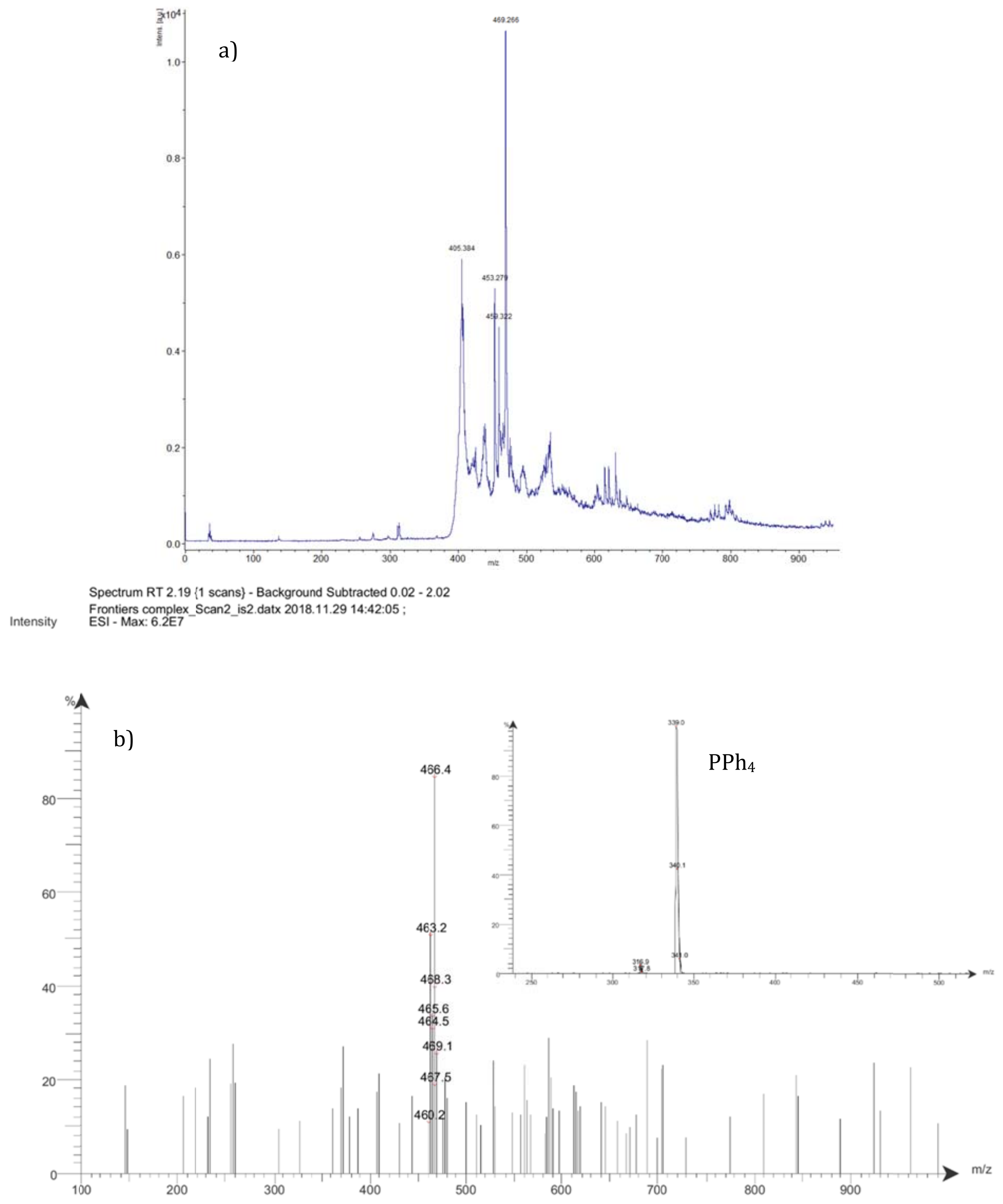

Figure S19. (a) MALDI-TOF-MS spectrum of complex **5** obtained in negative ion linear mode using 2,5-dihydroxy benzoic acid (DHB) as matrix (thin layer technique). (b) ESI-MS (-) spectrum (high temperature/medium fragmentation) of the complex **5** in methanol. Insert: ESI-MS (+) spectrum of **5**.

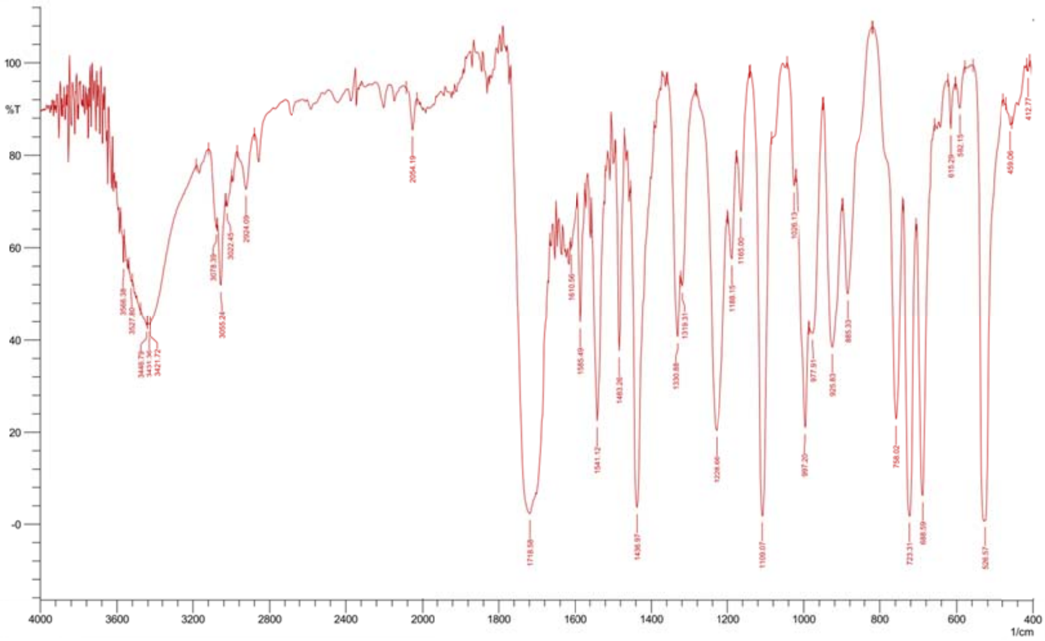

Figure S20. FT-IR spectra of $(PPh_4)_2[MoO(mohdt)_2]$ (**5**) in KBr pellet at 25 °C.

Nr. Name	C [%]	H [%]	N [%]	S [%]
MA-CHNF5	60.79	4.377	0.16	11.129
MA-CHNF5	60.45	4.351	0.13	11.048
MA-CHNF5	60.87	4.366	0.13	11.104
MA-CHNF5 (average)	**60.70**	**4.354**	**0.14**	**11.10**

Nr. Name	C [%]	H [%]	N [%]	S [%]
17 MA-CHNF5	60.79	4.377	0.16	11.129
20 MA-CHNF5	60.45	4.351	0.13	11.048
21 MA-CHNF5	60.87	4.366	0.13	11.104
Mittelwert	60.70	4.365	0.14	11.093

Figure S21. Elemental analysis of complex **5**. Calc. (%): C, 60.62; H, 4.74; S, 11.16.

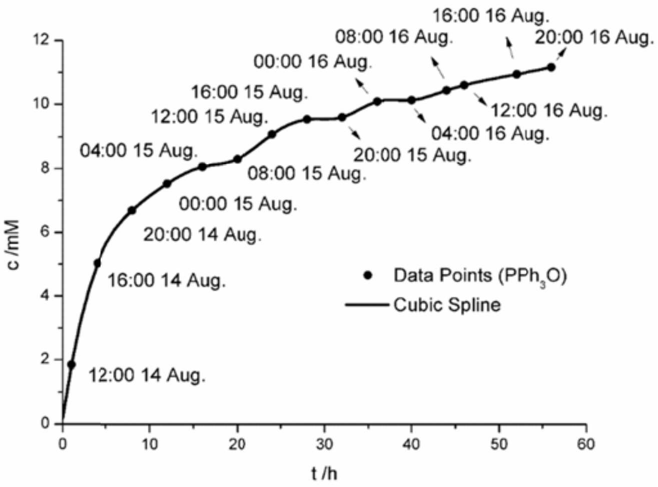

Figure S22. Increasing [PPh_3O] (mM) plotted against time (h) for the OAT reaction between DMSO and PPh_3 catalyzed by 5. Conditions: [PPh_3]0 = 9 mM; [catalyst] = 3 mM in DMSO (0.5 mL) at r.t.; concentration of PPh_3O was monitored by ^{31}P NMR spectroscopy every 4 hours (the times of day/night for each data point are indicated relating to the respective intensity of sunlight/temperature in the NMR-lab). The fitting procedure, which gave the best fit (cubic spline) was applied with routines as implemented in MatLab according to the book: A Practical Guide to Splines by Carl de Boor, Springer-Verlag New York, 1978. MatLab is using polynomials of degree 3 with special "not a knot" boundary conditions at both ends. In our special case we had 15 knots which we interpolated to 100 equidistant points to obtain a smooth line which is supposed to help the viewer to see the daytime dependence of the data points. The interpolation does by no means provide any kinetic information.

Notably, the resulting graph from evaluating the ^{31}P-NMR data for the catalysis as shown in Figure S22 shows some considerable yet consistent noise (wave like development after ca. 10 h). At first this observation was very puzzling until we eventually realized that the NMR data was collected at r.t., i.e. without temperature control, that the measurements were run automatically over the weekend (Aug. 15th/16th) after having been started on Friday Aug. 14th, i.e. without sufficient air conditioning in the lab for two days, that the lab is exposed to intense sun light in the morning and that it was a rather hot and cloudless weekend, which we selected for this long time run. The changes in reaction rate are therefore most likely due to the changing temperature in the lab and the reactions slowed down overnight and significantly accelerated in the morning up to approximately 2 p.m. Due to the resulting poor data to noise ratio and the slowness of the overall reaction we abstained from calculating any kinetic parameters from this experiment.

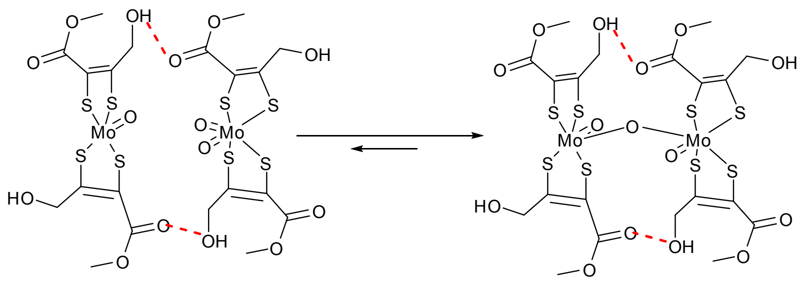

Figure S23. Plausible hydrogen bonding interaction between monomers and dimer formation.

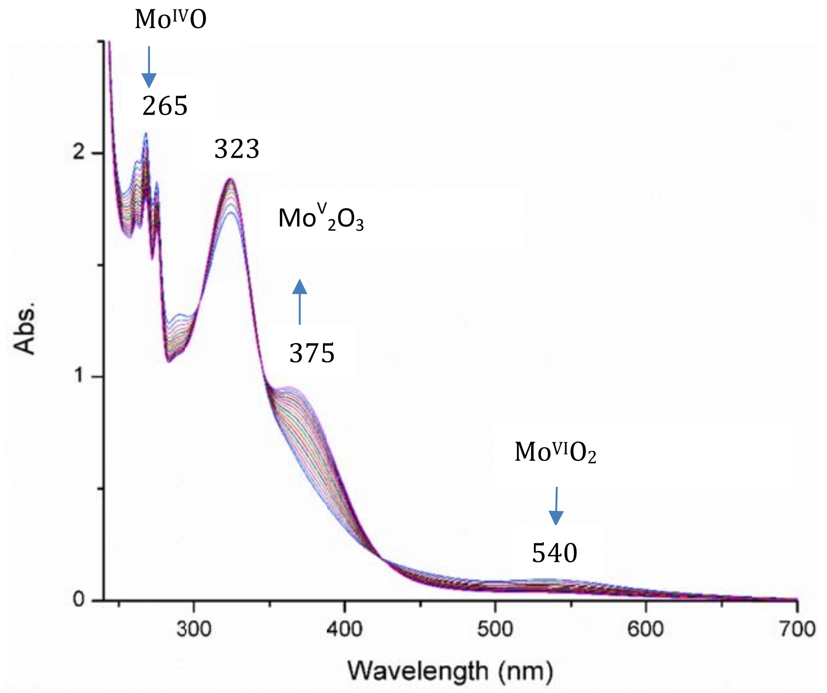

$[Mo^{IV}O(mohdt)_2]^{2-} + Me_3NO \longrightarrow [Mo^{VI}O_2(mohdt)_2]^{2-} + Me_3N$ (a)

$[Mo^{VI}O_2(mohdt)_2]^{2-} + [Mo^{IV}O(mohdt)_2]^{2-} \rightleftharpoons [Mo^{V}{}_2O_3(mohdt)_4]^{4-}$ (b)

Figure S24. Changes in the electronic spectrum of an acetonitrile solution of $[Mo^{IV}O(mohdt)_2]^{2-}$ (5) (0.3 mM) in the presence of trimethylaminoxid (TMAO). Note: the band at 323 nm already present in the Mo^{IV} species is in fact raised while all other signals of complex **5** decrease in intensity; taking into account the cumulative nature of UV-vis spectra this supports its tentative assignment as LLCT band.

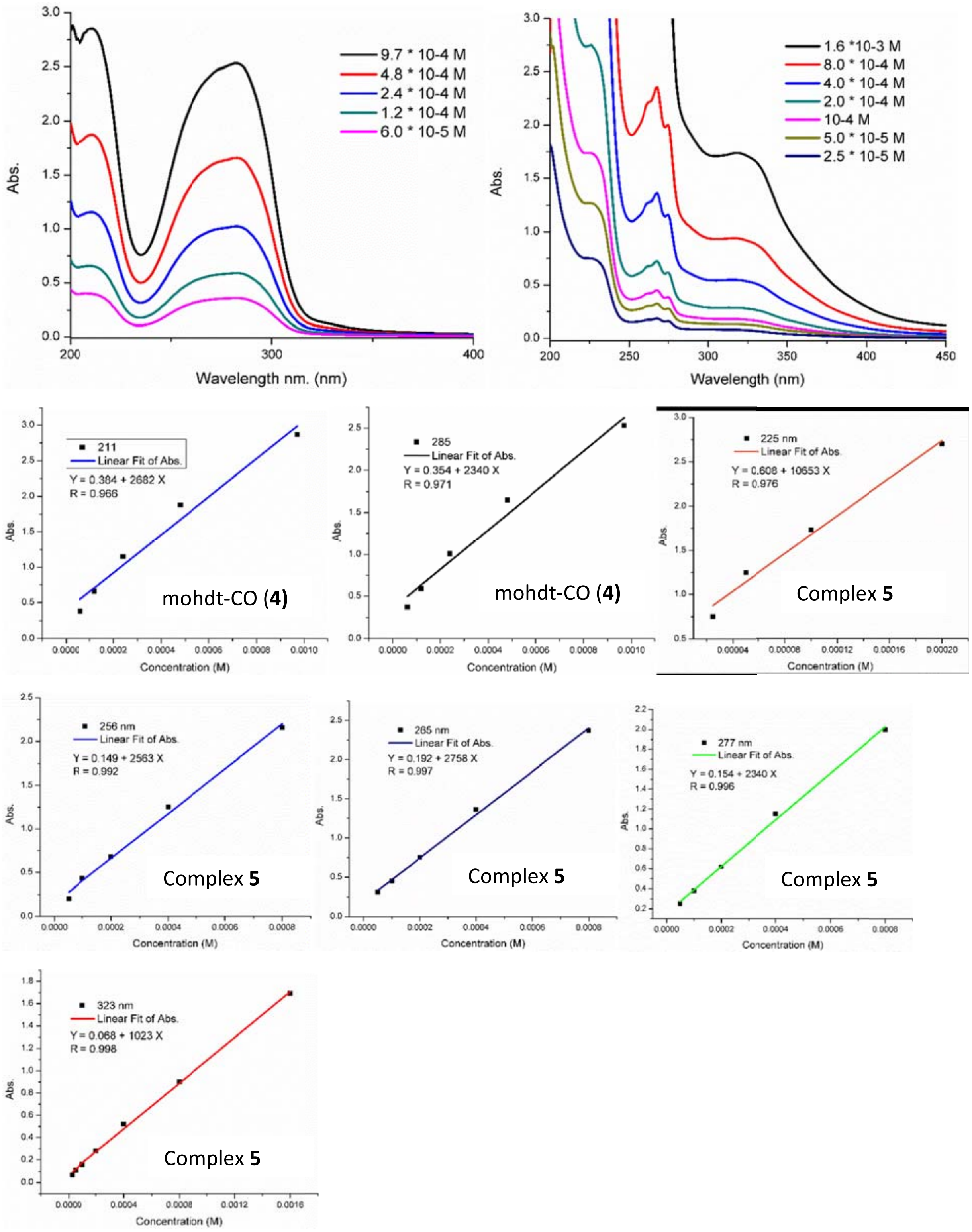

Figure S25. UV-visible spectra of mohdt-CO **4** (top left) and **5** (top right) in acetonitrile in various concentration; Extinction coefficient diagram at corresponding λ_{max} (bottom).

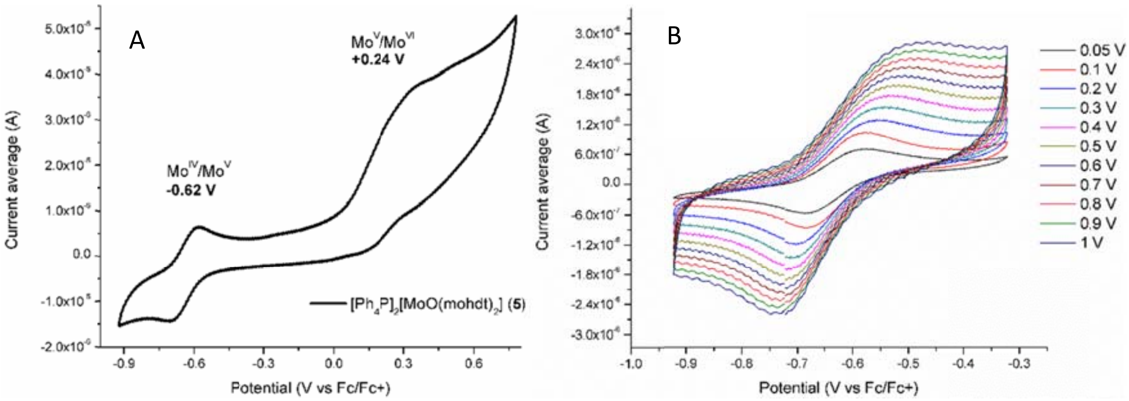

Figure S26. A) Cyclic voltammogram of 1 mM solution of **5** (left) in CH_3CN containing 0.1 M of Bu_4NPF_6 as electrolyte (scan rate: 0.1 Vs^{-1}). B) Cyclic voltammogram of **5** at different scan rates for the reversible redox couple Mo^{IV}/Mo^{V} (right). The peak potentials were recorded vs. internal reference $[Fc]/[Fc]^{+}$ at 298 K.

Structural Characterization

Table S1. Atomic coordinates (x 10^4) and equivalent isotropic displacement parameters (A^2 x 10^3) for **1**. U(eq) is defined as one third of the trace of the orthogonalized Uij tensor.

	x	y	z	U(eq)
C(1)	487(2)	2431(3)	8983(3)	41(1)
C(2)	2073(2)	3850(3)	8470(4)	41(1)
C(3)	2360(2)	2489(3)	8467(4)	40(1)
C(4)	2691(2)	5076(3)	8113(4)	44(1)
C(5)	3078(3)	7453(3)	8808(5)	66(1)
C(6)	3425(2)	2017(3)	8378(4)	45(1)
C(7)	4403(3)	234(4)	7452(5)	63(1)
O(1)	3167(2)	5106(2)	7007(3)	62(1)
O(2)	2598(2)	6132(2)	9125(3)	53(1)
O(3)	4189(2)	2669(3)	8944(4)	78(1)
O(4)	3403(2)	782(2)	7621(3)	53(1)
S(1)	-634(1)	1983(1)	9374(1)	57(1)
S(2)	838(1)	4168(1)	8786(1)	47(1)
S(3)	1444(1)	1248(1)	8734(1)	45(1)

Table S2. Bond lengths [Å] and angles [°] for **1**.

C(1)-S(1)	1.636(3)
C(1)-S(2)	1.727(3)
C(1)-S(3)	1.732(3)
C(2)-C(3)	1.345(4)
C(2)-C(4)	1.484(4)
C(2)-S(2)	1.730(3)
C(3)-C(6)	1.492(4)
C(3)-S(3)	1.734(3)
C(4)-O(1)	1.199(4)
C(4)-O(2)	1.316(3)
C(5)-O(2)	1.452(4)
C(6)-O(3)	1.189(4)
C(6)-O(4)	1.319(4)
C(7)-O(4)	1.453(4)
S(1)-C(1)-S(2)	122.50(18)
S(1)-C(1)-S(3)	124.59(18)
S(2)-C(1)-S(3)	112.90(16)
C(3)-C(2)-C(4)	125.7(3)
C(3)-C(2)-S(2)	116.3(2)
C(4)-C(2)-S(2)	117.9(2)
C(2)-C(3)-C(6)	123.9(3)
C(2)-C(3)-S(3)	116.5(2)
C(6)-C(3)-S(3)	119.5(2)
O(1)-C(4)-O(2)	125.5(3)
O(1)-C(4)-C(2)	124.2(3)
O(2)-C(4)-C(2)	110.2(3)
O(3)-C(6)-O(4)	125.0(3)
O(3)-C(6)-C(3)	123.9(3)
O(4)-C(6)-C(3)	111.2(3)
C(4)-O(2)-C(5)	116.3(3)
C(6)-O(4)-C(7)	115.5(3)
C(1)-S(2)-C(2)	97.34(14)
C(1)-S(3)-C(3)	96.97(14)

Symmetry transformations used to generate equivalent atoms:

Table S3. Anisotropic displacement parameters (A^2 x 10^3) for **1**. The anisotropic displacement factor exponent takes the form: -2 pi^2 [h^2 a*^2 U11 + ... + 2 h k a* b* U12]

	U11	U22	U33	U23	U13	U12
C(1)	40(2)	46(2)	38(2)	1(1)	8(1)	-3(1)
C(2)	34(1)	41(2)	47(2)	-1(1)	11(1)	-3(1)
C(3)	33(1)	38(2)	49(2)	1(1)	8(1)	0(1)
C(4)	36(2)	40(2)	57(2)	2(1)	11(1)	-2(1)
C(5)	74(3)	45(2)	80(2)	-8(2)	19(2)	-19(2)
C(6)	35(2)	40(2)	60(2)	1(1)	8(1)	-1(1)
C(7)	50(2)	56(2)	88(3)	2(2)	27(2)	14(2)
O(1)	58(2)	54(1)	83(2)	-7(1)	38(1)	-12(1)
O(2)	60(1)	37(1)	64(1)	-4(1)	21(1)	-9(1)
O(3)	35(1)	64(2)	128(2)	-28(2)	4(1)	-4(1)
O(4)	39(1)	45(1)	79(2)	-8(1)	21(1)	0(1)
S(1)	44(1)	59(1)	73(1)	-2(1)	26(1)	-11(1)
S(2)	40(1)	40(1)	66(1)	2(1)	20(1)	1(1)
S(3)	38(1)	37(1)	61(1)	3(1)	13(1)	-3(1)

Table S4. Hydrogen bonds for **1** [Å and °].

Nr	Typ	Res	Donor --- H....Acceptor	[ARU]	D - H	H...A	D...A	D - H...A
1		1	C(5) --H(5B) ..O(1)	[4545.01]	0.96	2.52	3.458(4)	165

Table S5. Atomic coordinates (x 10^4) and equivalent isotropic displacement parameters (A^2 x 10^3) for 2. U(eq) is defined as one third of the trace of the orthogonalized Uij tensor.

	x	y	z	U(eq)
C(1)	1801(3)	6684(9)	5039(2)	64(1)
C(2)	5489(4)	-201(11)	6355(4)	85(1)
C(3)	3735(3)	1372(8)	6486(2)	62(1)
C(4)	2871(3)	3205(8)	6107(2)	59(1)
C(5)	1928(3)	3523(8)	6438(2)	57(1)
C(6)	1616(3)	2275(9)	7266(2)	63(1)
O(1)	4591(2)	1563(7)	6041(2)	73(1)
O(2)	3680(2)	-122(7)	7111(2)	80(1)
O(3)	563(2)	3094(6)	7401(2)	65(1)
S(1)	1393(1)	8860(3)	4250(1)	77(1)
S(2)	3036(1)	5090(2)	5154(1)	66(1)
S(3)	1012(1)	5714(2)	5856(1)	61(1)

Table S6. Bond lengths [Å] and angles [°] for **2**.

C(1)-S(1)	1.632(4)
C(1)-S(2)	1.718(4)
C(1)-S(3)	1.740(4)
C(2)-O(1)	1.443(5)
C(3)-O(2)	1.196(5)
C(3)-O(1)	1.337(4)
C(3)-C(4)	1.461(5)
C(4)-C(5)	1.346(5)
C(4)-S(2)	1.746(4)
C(5)-C(6)	1.497(5)
C(5)-S(3)	1.729(4)
C(6)-O(3)	1.415(4)
S(1)-C(1)-S(2)	124.3(2)
S(1)-C(1)-S(3)	122.9(2)
S(2)-C(1)-S(3)	112.8(2)
O(2)-C(3)-O(1)	124.5(4)
O(2)-C(3)-C(4)	124.2(3)
O(1)-C(3)-C(4)	111.3(3)
C(5)-C(4)-C(3)	124.3(3)
C(5)-C(4)-S(2)	116.5(3)
C(3)-C(4)-S(2)	119.2(3)
C(4)-C(5)-C(6)	126.6(3)
C(4)-C(5)-S(3)	115.9(3)
C(6)-C(5)-S(3)	117.5(3)
O(3)-C(6)-C(5)	110.3(3)
C(3)-O(1)-C(2)	115.8(3)
C(1)-S(2)-C(4)	97.20(18)
C(5)-S(3)-C(1)	97.63(18)

Symmetry transformations used to generate equivalent atoms:

Table S7. Anisotropic displacement parameters (A^2 x 10^3) for **2**. The anisotropic displacement factor exponent takes the form: -2 pi^2 [h^2 a*^2 U11 + ... + 2 h k a* b* U12]

	U11	U22	U33	U23	U13	U12
C(1)	66(2)	63(2)	63(2)	-4(2)	10(2)	-10(2)
C(2)	68(2)	91(3)	99(3)	3(3)	15(2)	17(2)
C(3)	61(2)	62(2)	63(2)	-3(2)	9(2)	1(2)
C(4)	59(2)	57(2)	63(2)	-2(2)	12(2)	-2(2)
C(5)	62(2)	53(2)	57(2)	-8(1)	10(1)	-5(2)
C(6)	61(2)	72(2)	59(2)	-2(2)	14(2)	1(2)
O(1)	59(1)	82(2)	80(2)	6(1)	19(1)	7(1)
O(2)	77(2)	92(2)	74(2)	16(2)	17(1)	14(2)
O(3)	61(1)	61(2)	75(2)	-2(1)	22(1)	-2(1)
S(1)	79(1)	82(1)	71(1)	16(1)	4(1)	-7(1)
S(2)	62(1)	73(1)	64(1)	5(1)	16(1)	-4(1)
S(3)	59(1)	64(1)	62(1)	2(1)	10(1)	0(1)

Table S8. Hydrogen coordinates (x 10^4) and isotropic displacement parameters (A^2 x 10^3) for **2**.

	x	y	z	U(eq)
H(2A)	5734	439	6921	128
H(2B)	6049	20	5980	128
H(2C)	5283	-2237	6372	128
H(6A)	1672	141	7254	76
H(6B)	2094	3001	7735	76
H(3O)	200(40)	1540(130)	7370(40)	96(17)

Table S9. Hydrogen bonds for **2** [Å and °].

Nr	Typ	Res	Donor --- H....Acceptor	[ARU]	D - H	H...A	D...A	D - H...A
1		1	O(3) --H(3O) ..O(3)	[2555.01]	0.84(6)	1.89(6)	2.708(4)	166(6)
2		1	C(2) --H(2A) ..O(2)	[2645.01]	0.96	2.59	3.430(7)	146

Translation of ARU-Code to CIF and Equivalent Position Code

[2645.] = 1-x,-1/2+y,1/2-z
[2555.] = -x,1/2+y,1/2-z

Table S10. Atomic coordinates (x 10^4) and equivalent isotropic displacement parameters (A^2 x 10^3) for **3**. U(eq) is defined as one third of the trace of the orthogonalized Uij tensor.

	x	y	z	U(eq)
S(1)	2853(1)	4555(1)	3946(1)	26(1)
S(2)	4912(1)	2887(1)	4815(1)	26(1)
S(3)	3290(1)	4357(1)	6002(1)	31(1)
O(1)	2232(2)	4740(2)	1996(2)	29(1)
O(2)	6836(2)	1853(2)	3677(2)	31(1)
C(1)	3314(3)	3867(3)	2180(2)	28(1)
C(2)	3710(3)	3686(3)	3208(2)	22(1)
C(3)	4686(3)	2924(3)	3610(2)	24(1)
C(4)	5554(3)	2094(3)	3108(2)	30(1)
C(5)	3666(3)	3961(3)	4967(2)	25(1)
S(4)	1520(1)	1409(1)	4734(1)	29(1)
S(5)	1259(1)	1056(1)	2770(1)	28(1)
S(6)	3363(1)	-386(1)	4002(1)	37(1)
O(3)	-1331(3)	2697(2)	5157(2)	34(1)
O(4)	-26(2)	3240(2)	1727(2)	29(1)
C(6)	-419(3)	3251(3)	4593(2)	29(1)
C(7)	314(3)	2334(3)	4071(2)	23(1)
C(8)	163(3)	2151(3)	3150(2)	23(1)
C(9)	-807(3)	2772(3)	2415(2)	26(1)
C(10)	2113(3)	627(3)	3839(2)	26(1)

Table S11. Bond lengths [Å] and angles [°] for **3**.

S(1)-C(5)	1.718(3)
S(1)-C(2)	1.736(3)
S(2)-C(5)	1.727(3)
S(2)-C(3)	1.741(3)
S(3)-C(5)	1.659(3)
O(1)-C(1)	1.422(4)
O(2)-C(4)	1.418(4)
C(1)-C(2)	1.510(4)
C(2)-C(3)	1.339(4)
C(3)-C(4)	1.501(4)
S(4)-C(10)	1.728(3)
S(4)-C(7)	1.743(3)
S(5)-C(10)	1.728(3)
S(5)-C(8)	1.748(3)
S(6)-C(10)	1.641(3)
O(3)-C(6)	1.423(4)
O(4)-C(9)	1.431(3)
C(6)-C(7)	1.502(4)
C(7)-C(8)	1.347(4)
C(8)-C(9)	1.493(4)
C(5)-S(1)-C(2)	97.17(14)
C(5)-S(2)-C(3)	96.88(15)
O(1)-C(1)-C(2)	111.0(2)
C(3)-C(2)-C(1)	125.9(3)
C(3)-C(2)-S(1)	116.4(2)
C(1)-C(2)-S(1)	117.7(2)
C(2)-C(3)-C(4)	125.3(3)
C(2)-C(3)-S(2)	116.2(2)
C(4)-C(3)-S(2)	118.5(2)
O(2)-C(4)-C(3)	109.3(3)
S(3)-C(5)-S(1)	123.85(17)
S(3)-C(5)-S(2)	122.78(19)
S(1)-C(5)-S(2)	113.36(17)
C(10)-S(4)-C(7)	98.06(15)
C(10)-S(5)-C(8)	98.03(15)
O(3)-C(6)-C(7)	111.8(2)
C(8)-C(7)-C(6)	127.8(3)
C(8)-C(7)-S(4)	116.0(2)
C(6)-C(7)-S(4)	116.2(2)
C(7)-C(8)-C(9)	128.3(3)
C(7)-C(8)-S(5)	115.7(2)
C(9)-C(8)-S(5)	116.1(2)
O(4)-C(9)-C(8)	109.4(2)
S(6)-C(10)-S(4)	123.1(2)
S(6)-C(10)-S(5)	124.71(19)
S(4)-C(10)-S(5)	112.18(16)

Symmetry transformations used to generate equivalent atoms:

Table S12. Anisotropic displacement parameters (A^2 x 10^3) for **3**. The anisotropic displacement factor exponent takes the form: -2 pi^2 [h^2 a*^2 U11 + ... + 2 h k a* b* U12].

	U11	U22	U33	U23	U13	U12
S(1)	24(1)	26(1)	27(1)	1(1)	4(1)	4(1)
S(2)	24(1)	28(1)	27(1)	1(1)	3(1)	4(1)
S(3)	33(1)	35(1)	27(1)	1(1)	9(1)	5(1)
O(1)	23(1)	28(1)	34(1)	5(1)	-2(1)	0(1)
O(2)	25(1)	33(1)	34(1)	-8(1)	-3(1)	8(1)
C(1)	28(2)	27(2)	29(2)	1(1)	4(1)	1(1)
C(2)	21(1)	21(1)	24(2)	1(1)	1(1)	-4(1)
C(3)	22(2)	23(2)	26(2)	-1(1)	3(1)	-2(1)
C(4)	24(2)	34(2)	32(2)	-6(2)	2(1)	7(1)
C(5)	20(1)	25(2)	29(2)	3(1)	4(1)	0(1)
S(4)	27(1)	36(1)	24(1)	2(1)	2(1)	5(1)
S(5)	30(1)	28(1)	26(1)	0(1)	8(1)	4(1)
S(6)	28(1)	33(1)	51(1)	5(1)	7(1)	9(1)
O(3)	26(1)	52(2)	24(1)	-6(1)	4(1)	-1(1)
O(4)	28(1)	37(1)	23(1)	6(1)	4(1)	-6(1)
C(6)	28(2)	30(2)	29(2)	0(1)	3(1)	4(1)
C(7)	18(1)	25(2)	26(2)	3(1)	4(1)	-2(1)
C(8)	20(1)	23(2)	27(2)	-1(1)	8(1)	0(1)
C(9)	24(2)	33(2)	23(2)	5(1)	4(1)	-3(1)
C(10)	22(1)	23(2)	34(2)	1(1)	7(1)	-1(1)

Table S13. Hydrogen coordinates (x 10^4) and isotropic displacement parameters (A^2 x 10^3) for **3**.

	x	y	z	U(eq)
H(1A)	4145	4137	1906	34
H(1B)	2996	3091	1889	34
H(4A)	5043	1330	2955	36
H(4B)	5740	2474	2522	36
H(1O)	1530(40)	4380(30)	1870(30)	42(11)
H(2O)	7150(40)	1180(30)	3530(30)	53(12)
H(6A)	287	3738	4987	35
H(6B)	-965	3802	4147	35
H(9A)	-1520	2194	2127	32
H(9B)	-1295	3438	2690	32
H(3O)	-1860(40)	2380(30)	4880(30)	38(13)
H(4O)	-470(40)	3070(40)	1240(30)	63(15)

Table S14. Hydrogen bonds for **3** [A and deg.].

Nr	Typ	Res	Donor --- H....Acceptor	[ARU]	D - H	H...A	D...A	D - H...A
1		1	O(1) --H(1O) ..O(4)	[ ]	0.79(4)	1.95(4)	2.727(3)	168(3)
2		1	O(2) --H(2O) ..O(1)	[2645.01]	0.84(3)	1.90(4)	2.737(3)	171(4)
3		2	O(3) --H(3O) ..O(2)	[1455.01]	0.70(4)	2.10(4)	2.762(3)	158(4)
4		2	O(4) --H(4O) ..O(3)	[4554.02]	0.80(4)	1.89(4)	2.669(3)	166(4)

Translation of ARU-Code to CIF and Equivalent Position Code

[4554.] =	x,1/2-y,-1/2+z
[2645.] =	1-x,-1/2+y,1/2-z
[1455.] =	-1+x,y,z

Table S15. Atomic coordinates (x 10^4) and equivalent isotropic displacement parameters (A^2 x 10^3) for **4**. U(eq) is defined as one third of the trace of the orthogonalized Uij tensor.

	x	y	z	U(eq)
S(1)	4076(1)	7219(1)	1673(1)	26(1)
S(2)	7059(1)	5941(1)	616(1)	23(1)
O(4)	9210(4)	2992(2)	699(1)	28(1)
O(1)	4751(4)	8634(2)	669(1)	37(1)
O(3)	7999(4)	2505(2)	1639(1)	36(1)
O(2)	2925(4)	5564(2)	2681(1)	38(1)
C(4)	6767(4)	4837(2)	1243(1)	22(1)
C(3)	5409(4)	5422(2)	1727(1)	23(1)
C(5)	8034(5)	3330(2)	1223(1)	23(1)
C(1)	5227(5)	7499(2)	931(1)	26(1)
C(6)	10519(6)	1515(2)	640(1)	33(1)
C(2)	4922(5)	4688(2)	2317(1)	27(1)

Table S16. Bond lengths [Å] and angles [°] for **4**.

S(1)-C(3)	1.727(2)
S(1)-C(1)	1.776(2)
S(2)-C(4)	1.7463(19)
S(2)-C(1)	1.760(2)
O(4)-C(5)	1.328(2)
O(4)-C(6)	1.454(2)
O(1)-C(1)	1.204(2)
O(3)-C(5)	1.206(2)
O(2)-C(2)	1.412(2)
C(4)-C(3)	1.349(3)
C(4)-C(5)	1.466(3)
C(3)-C(2)	1.512(3)
C(3)-S(1)-C(1)	96.54(9)
C(4)-S(2)-C(1)	95.58(9)
C(5)-O(4)-C(6)	115.92(15)
C(3)-C(4)-C(5)	123.06(17)
C(3)-C(4)-S(2)	118.10(15)
C(5)-C(4)-S(2)	118.84(13)
C(4)-C(3)-C(2)	127.55(18)
C(4)-C(3)-S(1)	116.75(15)
C(2)-C(3)-S(1)	115.69(14)
O(3)-C(5)-O(4)	124.51(18)
O(3)-C(5)-C(4)	123.18(17)
O(4)-C(5)-C(4)	112.30(16)
O(1)-C(1)-S(2)	123.79(16)
O(1)-C(1)-S(1)	123.17(16)
S(2)-C(1)-S(1)	113.03(11)
O(2)-C(2)-C(3)	111.10(17)

Symmetry transformations used to generate equivalent atoms:

Table S17. Anisotropic displacement parameters (A^2 x 10^3) for **4**. The anisotropic displacement factor exponent takes the form: -2 pi^2 [h^2 a*^2 U11 + ... + 2 h k a* b* U12]

	U11	U22	U33	U23	U13	U12
S(1)	26(1)	23(1)	27(1)	-6(1)	3(1)	1(1)
S(2)	29(1)	21(1)	21(1)	0(1)	2(1)	1(1)
O(4)	39(1)	20(1)	26(1)	-2(1)	8(1)	5(1)
O(1)	47(1)	23(1)	41(1)	5(1)	5(1)	6(1)
O(3)	51(1)	27(1)	30(1)	6(1)	8(1)	8(1)
O(2)	45(1)	41(1)	29(1)	-12(1)	14(1)	-7(1)
C(4)	22(1)	21(1)	21(1)	-2(1)	1(1)	-2(1)
C(3)	20(1)	23(1)	24(1)	-2(1)	0(1)	-3(1)
C(5)	23(1)	22(1)	24(1)	-1(1)	1(1)	0(1)
C(1)	26(1)	22(1)	31(1)	-2(1)	0(1)	-1(1)
C(6)	38(1)	22(1)	38(1)	-5(1)	10(1)	5(1)
C(2)	30(1)	31(1)	22(1)	-1(1)	4(1)	-3(1)

Table S18. Hydrogen coordinates (x 10^4) and isotropic displacement parameters (A^2 x 10^3) for **4**.

	x	y	z	U(eq)
H(6A)	12219	1313	966	49
H(6B)	11604	1418	260	49
H(6C)	8615	817	655	49
H(2A)	3770	3734	2249	33
H(2B)	7196	4505	2520	33
H(3O)	4260(80)	6010(40)	2894(15)	72(11)

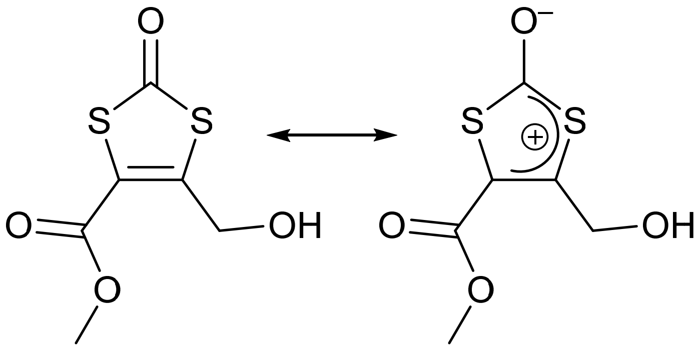

Figure S27: Proposed resonance forms of compound **4**. While the left resonance form is certainly the predominant one, metrical parameters for the C-O, C-S and C-C bonds indicate a stronger push of electrons from the alcohol substituted molecule side to the C=O moiety in **4** compared to the other complexes which bear a C=S moiety.
